# PrEP uptake, persistence, adherence, and effect of retrospective drug level feedback on PrEP adherence among young women in southern Africa: Results from HPTN 082, a randomized controlled trial

**DOI:** 10.1371/journal.pmed.1003670

**Published:** 2021-06-18

**Authors:** Connie Celum, Sybil Hosek, Mandisa Tsholwana, Sheetal Kassim, Shorai Mukaka, Bonnie J. Dye, Subash Pathak, Nyaradzo Mgodi, Linda-Gail Bekker, Deborah J. Donnell, Ethan Wilson, Krista Yuha, Peter L. Anderson, Yaw Agyei, Heather Noble, Scott M. Rose, Jared M. Baeten, Jessica M. Fogel, Adeola Adeyeye, Lubbe Wiesner, James Rooney, Sinead Delany-Moretlwe

**Affiliations:** 1 Departments of Global Health, Medicine, and Epidemiology, University of Washington, Seattle, Washington, United States of America; 2 Department of Psychiatry, Stroger Hospital of Cook County, Chicago, Illinois, United States of America; 3 Wits RHI, University of Witwatersrand, Johannesburg, South Africa; 4 Desmond Tutu HIV Centre, University of Cape Town, Cape Town, South Africa; 5 University of Zimbabwe, College of Health Sciences Clinical Trials Unit Research Unit, Harare, Zimbabwe; 6 Family Health International 360, Durham, North Carolina, United States of America; 7 Vaccine and Infectious Disease Division, Fred Hutchinson Cancer Research Center, Seattle, Washington, United States of America; 8 Department of Pharmaceutical Sciences, University of Colorado, Aurora, Colorado, United States of America; 9 Department of Pathology, Johns Hopkins University School of Medicine, Baltimore, Maryland, United States of America; 10 National Institutes of Allergy and Infectious Diseases, National Institutes of Health, Division of AIDS, Rockville, Maryland, United States of America; 11 Division of Clinical Pharmacology, Department of Medicine, University of Cape Town, Cape Town, South Africa; 12 Gilead Sciences, Foster City, California, United States of America; UMICH, UNITED STATES

## Abstract

**Background:**

Pre-exposure prophylaxis (PrEP) is highly effective and an important prevention tool for African adolescent girls and young women (AGYW), but adherence and persistence are challenging. PrEP adherence support strategies for African AGYW were studied in an implementation study.

**Methods and findings:**

HIV Prevention Trials Network (HPTN) 082 was conducted in Cape Town, Johannesburg (South Africa) and Harare (Zimbabwe) from October 2016 to October 2018 to evaluate PrEP uptake, persistence, and the effect of drug level feedback on adherence. Sexually active HIV–negative women ages 16–25 were offered PrEP and followed for 12 months; women who accepted PrEP were randomized to standard adherence support (counseling, 2-way SMS, and adherence clubs) or enhanced adherence support with adherence feedback from intracellular tenofovir-diphosphate (TFV-DP) levels in dried blood spots (DBS). PrEP uptake, persistence through 12 months (no PrEP hold or missed visits), and adherence were assessed. The primary outcome was high adherence (TFV-DP ≥700 fmol/punch) at 6 months, compared by study arm. Of 451 women enrolled, median age was 21 years, and 39% had curable sexually transmitted infections (STIs). Most (95%) started PrEP, of whom 55% had uninterrupted PrEP refills through 12 months. Of those with DBS, 84% had detectable TFV-DP levels at month 3, 57% at month 6, and 31% at month 12. At 6 months, 36/179 (21%) of AGYW in the enhanced arm had high adherence and 40/184 (22%) in the standard adherence support arm (adjusted odds ratio [OR] of 0.92; 95% confidence interval [CI] 0.55, 1.34; *p* = 0.76). Four women acquired HIV (incidence 1.0/100 person-years), with low or undetectable TFV-DP levels at or prior to seroconversion, and none of whom had tenofovir or emtricitabine resistance mutations. The study had limited power to detect a modest effect of drug level feedback on adherence, and there was limited awareness of PrEP at the time the study was conducted.

**Conclusions:**

In this study, PrEP initiation was high, over half of study participants persisted with PrEP through month 12, and the majority of young African women had detectable TFV-DP levels through month 6 with one-fifth having high adherence. Drug level feedback in the first 3 months of PrEP use did not increase the proportion with high adherence at month 6. HIV incidence was 1% in this cohort with 39% prevalence of curable STIs and moderate PrEP adherence. Strategies to support PrEP use and less adherence-dependent formulations are needed for this population.

**Trial registration:**

ClinicalTrials.gov NCT02732730.

## Abstract 2

Connie Celum and co-workers report on use of pre-exposure prophylaxis by young women in Southern Africa.

## Author summary

**Why was this study done?:**

- HIV incidence remains high (4%) in young African women. Pre-exposure prophylaxis (PrEP) has over 90% efficacy in preventing HIV infection when used consistently and could reduce HIV incidence in young African women.
- Adherence was low in placebo-controlled efficacy trials of oral PrEP among young African women. Qualitative research among a subset of women at unblinding of the efficacy trials indicated that women thought drug level feedback would support their adherence and foster open discussions with counselors about adherence.
- Drug level feedback should be evaluated as a strategy to increase PrEP adherence among young African women.

**What did the researchers do and find?:**

- A cohort of 451 young women in South Africa and Zimbabwe who were at risk for HIV were recruited and offered PrEP, among whom 95% initiated PrEP.
- Half were randomized to receive drug level feedback at months 2 and 3 plus the standard adherence support package (2-way SMS, brief counseling, and optional PrEP adherence clubs), and half received the standard adherence support package without drug level feedback.
- The majority had detectable intracellular drug levels through the first 6 months, and 21% had high adherence at month 6, defined as intracellular tenofovir-diphosphate (TFV-DP) ≥700 fmol/punch. PrEP adherence did not differ based on whether they were randomized to receive drug level feedback at months 2 and 3 or to standard of care (SOC) PrEP adherence support without drug level feedback.

**What do these findings mean?:**

- Drug level feedback was not associated with improved PrEP adherence among young African women.
- Additional strategies need to be evaluated for their effectiveness in increasing adherence with oral PrEP for young African women.
- Longer-acting and less user-dependent PrEP formulations may also overcome adherence challenges with daily oral PrEP.

## Introduction

Adolescent girls and young women (AGYW) in sub-Saharan Africa account for approximately 25% of new HIV infections globally, and, in recent HIV prevention trials, have high HIV incidence despite monthly counseling and comprehensive prevention services [1–3]. The most efficacious biomedical HIV prevention intervention currently available is oral HIV pre-exposure prophylaxis (PrEP) with tenofovir/emtricitabine (TDF/FTC), which has high protection among those who use PrEP consistently, for both women and men [4].

Divergent results in the PrEP efficacy trials among African women were related to adherence; a meta-analysis of all PrEP trial results in women estimated 61% PrEP efficacy among women with >75% adherence based on plasma tenofovir levels [5]. In the VOICE and FEM-PrEP trials that did not demonstrate efficacy, retrospective drug level testing indicated that <25% of young African women randomized to TDF/FTC were adherent to their PrEP pills [6,7]. Qualitative research in VOICE and FEM-PrEP indicated reasons for low PrEP use included receiving a placebo or an investigational drug of unknown efficacy, fear of side effects, low research literacy, the need for social support, and fear of their partner’s reactions about product use [8–10]. Given the ongoing high HIV incidence among African AGYW, it is important to understand how to facilitate uptake and adherence to oral PrEP while in parallel developing and evaluating longer-acting and less adherence-dependent PrEP formulations.

Objective measures of adherence, such as drug levels, may assist counselors with providing individualized messages and directed feedback. After completion of the VOICE trial, a subset of women on the active arms received retrospective feedback about their drug levels during the trial, which they reported to be useful and suggested that real-time drug level monitoring and feedback would promote honest discussions about PrEP use [11]. A recent systematic review and meta-analysis of randomized controlled trials reported that feedback, based on subjective or objective measures of adherence, increased adherence by 10% [12]. Plasma tenofovir monitoring among men who have sex with men (MSM) in a United States demonstration project was used to trigger more intensive counseling support among those who had undetectable plasma tenofovir levels, reflecting no PrEP use in the prior week, and was associated with sustained improved PrEP adherence in 50% [13]. The effect of drug level feedback for African AGYW taking PrEP on subsequent adherence has not been evaluated. However, adherence counseling for persons living with HIV in South Africa using an objective marker (viral load) with same day feedback using a point-of-care viral load assay was associated with 14% higher rates of viral suppression [14].

HIV Prevention Trials Network (HPTN) 082 was designed as an open-label PrEP demonstration study among African AGYW with the following goals: (1) to understand PrEP use through evaluation of uptake, adherence, and persistence to PrEP over 12 months; and (2) to determine whether drug level feedback improved PrEP adherence in the initial 6 months after PrEP initiation. All women received an adherence support package with 2-way SMS, peer adherence support clubs, and brief counseling (standard adherence support). To assess impact of drug level feedback among women who initiated PrEP, we included a nested randomization to assess the impact of retrospective drug level feedback (enhanced adherence support) added to standard adherence support.

## Methods

### Study population

Between October 2016 and October 2018, HIV–negative women were recruited from Cape Town and Johannesburg, South Africa, and Harare, Zimbabwe (ClinicalTrials.gov NCT02732730). Women were eligible if they were literate, female at birth, ages 16 to 25 years, had vaginal or anal sex in the month prior to screening, and reported interest in taking PrEP in the 36-item HIV Prevention Readiness Measure about risk behaviors, medical care utilization, and PrEP beliefs (adapted from the HIV Treatment Readiness Measure) [15]. To screen out lower risk women, we required a VOICE rick score ≥5, which was associated with >6% HIV incidence in prior cohorts [16]. In addition, women needed to have regular access to a mobile phone with SMS capacity, be hepatitis B seronegative, and willing to accept hepatitis B virus (HBV) vaccine (if previous immunity was not confirmed), have normal renal function (creatinine clearance >60 ml/min), and not be pregnant. Study visits were at months 1, 2, 3, 6, 9, and 12 with HIV and pregnancy testing at every visit (S1 Text).

### Provision of PrEP and adherence support

Women were eligible to enroll regardless of their decision to initiate PrEP, and those who did not initiate PrEP at enrollment could initiate PrEP until month 9. A 1-month supply of PrEP was provided for the first 3 months, changing to a 3-month supply at month 3. Women could discontinue PrEP any time during the 12-month follow-up.

PrEP was held when women became pregnant or if creatinine clearance decreased by >10% from baseline or was <60 ml/min; women could restart PrEP after pregnancy and if their creatinine clearance returned to baseline. Women who had positive/reactive HIV tests had PrEP holds until confirmatory RNA tests were negative. Women who seroconverted to HIV were discontinued from PrEP, terminated from study follow-up, and referred for HIV care.

### Adherence support with nested randomized assessment of drug level feedback

When women initiated PrEP, at enrollment or a subsequent visit, women were randomized 1:1 to the standard or enhanced adherence support arm. Site-stratified randomization with block sizes of 2 and 4 was created by the study biostatistician, with web-based communication of arm allocation accessed by the site at the time of randomization. Participants and providers were not blinded to their intervention arm. Standard adherence support for PrEP was provided to both arms and included adherence counseling at each study visit (S2 Text), weekly 2-way text messages for the first 3 months, and voluntary participation in monthly adherence clubs.

**Fig 1:**
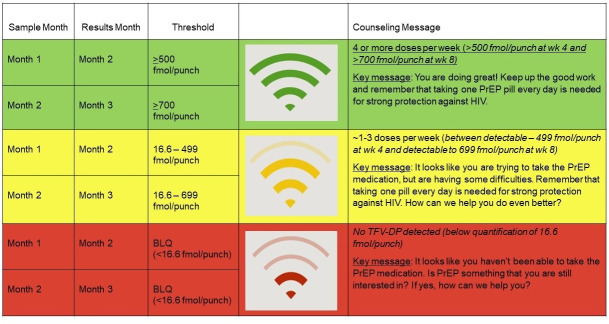
Drug level feedback based on semiquantitative measures of intracellular TFV-DP concentrations. BLQ, below the limit of quantification; PrEP, pre-exposure prophylaxis; TFV-DP, tenofovir-diphosphate.

Women in the enhanced adherence support received adherence feedback based on intracellular tenofovir-diphosphate (TFV-DP) levels in dried blood spots (DBS) twice during their first 3 months on PrEP, at month 2 (based on samples collected at month 1), and again at month 3 (based on samples collected at month 2). Drug level feedback was provided using a semiquantitative image of a wireless signal for high adherence (above the threshold for 4 or more doses per week on average, i.e., ≥500 fmol/punch at 4 weeks (pre-steady state) and ≥700 fmol/punch at 8 weeks (steady state) [17], medium (detectable but below the high adherence threshold; indicative of approximately 1 to 3 doses per week on average), and below the limit of quantitation (Fig 1). Counseling messages focused on encouraging medium to high adherence and problem-solving for those with undetectable levels.

### Laboratory methods

For drug level feedback, intracellular TFV-DP levels measured in DBS were performed at the University of Cape Town, which validated the DBS testing in collaboration with the University of Colorado, the developer of the TFV-DP DBS assay [18]. TFV-DP levels in DBS for endpoint analyses were performed at the University of Colorado. Intracellular TFV-DP in red blood cells has a 17-day half-life and 25-fold drug accumulation, which provides a cumulative measure of dosing and average adherence to PrEP in the prior 6 weeks [18]. Studies of directly observed PrEP dosing were used to define thresholds for DBS TFV-DP levels for categories of average weekly dosages taken of PrEP [17,19–21]. HIV status was determined by point-of-care rapid HIV tests at the site followed by an instrumented fourth generation antigen/antibody immunoassay (either GS IV Combo Ag/Ab EIA, Bio-Rad Laboratories, Hercules, California, USA or Architect HIV Ag/Ab Combo, Abbott Diagnostics, Wiesbaden, Germany) and RNA testing (Abbott RealTime HIV-1 Viral Load Assay, Abbott Molecular, Abbott Park, Illinois, USA) for acute infection for samples with positive rapid test or antigen/antibody immunoassays. HIV resistance testing on samples from seroconverters was performed using the ViroSeq HIV-1 Genotyping System v2.0 (Abbott Molecular, Des Plaines, Illinois, USA).

Creatinine clearance was estimated based on serum creatinine measurements using the Schwartz equation [19]. *Neisseria gonorrhoeae* and *Chlamydia trachomatis* were assessed by nucleic acid amplification (Cepheid GeneXpert, Sunnyvale, California, USA), and *Trichomonas vaginalis* by rapid test (OSOM Trichomonas Test, Sekisui Diagnostics, Burlington, Massachusetts, USA). Syphilis was assessed by rapid plasma reagin (Macro-Vue RPR Test, Becton Dickinson, Sparks, Maryland, USA) followed by a treponemal-specific confirmatory assay (Serodia-TPPA, Fujirebio, Tokyo, Japan or Randox-SYP-TPHA, Crumlin, County Antrim, United Kingdom).

### Ethical review

The study protocol was approved by the ethics review committees at each study site: University of Cape Town Faculty of Health Sciences (reference number: 129/2016), University of Witwatersrand, Human Research Ethics Committee (reference number: 160304), and University of Zimbabwe Joint Research Ethics Committee (reference Number: 27/16). All participants provided written informed consent in English or their local language. Following local regulations, participants below the legal age for consent provided assent, and parent or guardian informed consent was obtained.

### Endpoints

PrEP uptake was defined as the proportion of women enrolled who initiated PrEP by month 9. PrEP persistence was defined as time to first PrEP discontinuation, based on a PrEP clinical hold, elective PrEP stop, or missed visit (assuming all study pills were used by the date of first missed scheduled study visit). The primary endpoint for the enhanced adherence intervention was high adherence, defined as TFV-DP ≥700 fmol/punch, at month 6, with a secondary endpoint of high adherence at month 12.

### Sample size and design

The study planned to enroll 400 women who initiated PrEP, which had at least 80% power to detect a difference of 15% in the primary endpoint between the enhanced and standard adherence support arms at month 6, assuming 50% high adherence in the standard of care (SOC) arm and 10% loss to follow-up.

### Statistical analysis

**Fig 2:**
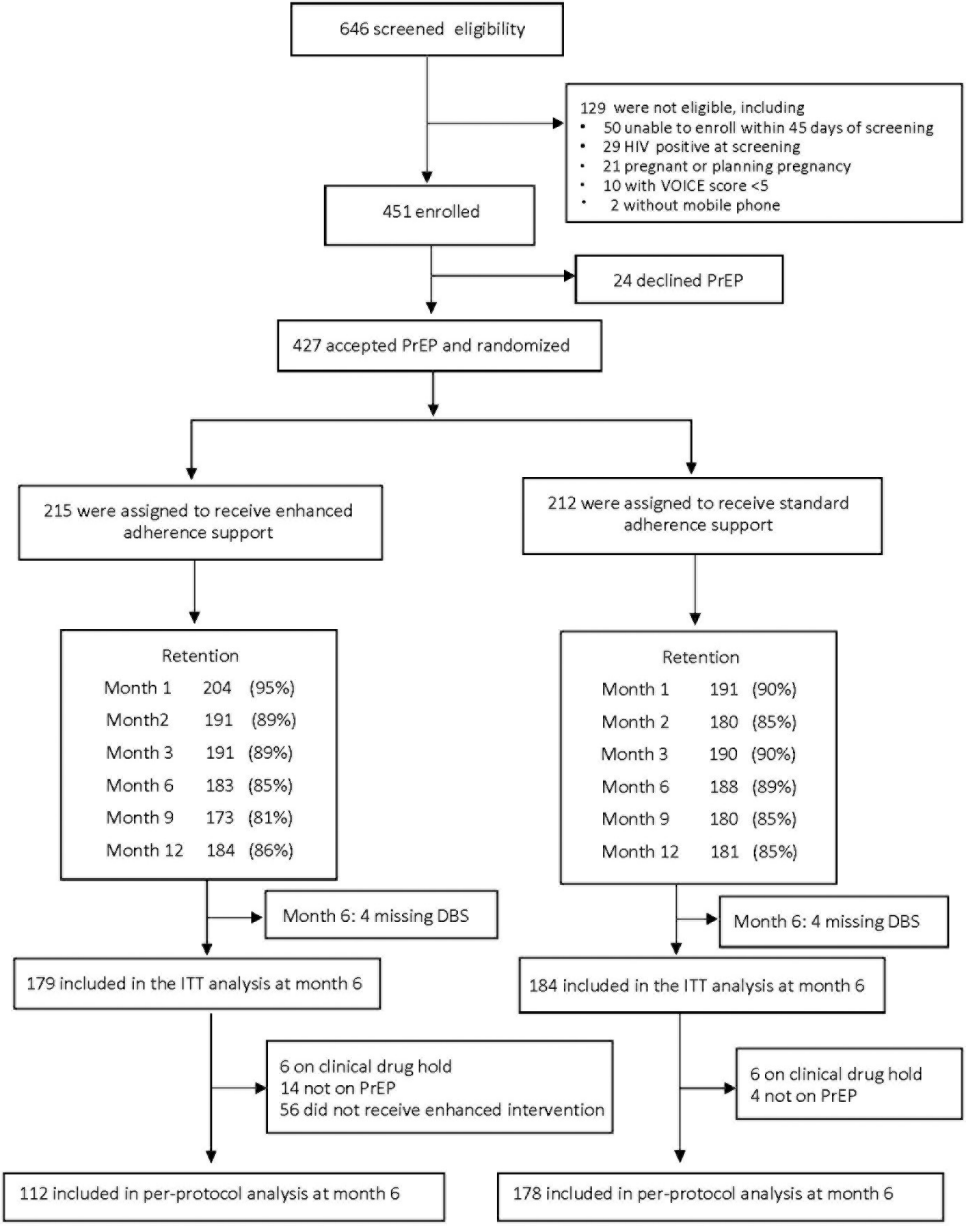
Participant flowchart diagram for HPTN 082. DBS, dried blood spots; HPTN, HIV Prevention Trials Network; ITT, intention-to-treat; PrEP, pre-exposure prophylaxis.

The study is reported according to the CONsolidated Standards of Reporting Trials (CONSORT) guidelines (S3 Text). The primary intention-to-treat analysis compared the proportion with high adherence (TFV-DP ≥700 fmol/punch) at month 6 in those randomized to standard versus enhanced adherence support using logistic regression, adjusted for site. A secondary analysis was linear regression evaluating high adherence (TFV-DP ≥700 fmol/punch) at 6 months in those randomized to standard versus enhanced adherence support, adjusted for site. A per-protocol analysis excluded data after women discontinued PrEP due to a clinical or laboratory hold and excluded women in the enhanced arm who did not fully receive the adherence feedback (either because DBS results were not returned before their next visit or because drug-level counseling did not correspond to the DBS drug levels from the laboratory). The participant flowchart summarizes the intention-to-treat and per-protocol cohorts (Fig 2, S3 Text). A Kaplan–Meier plot was used to assess differences in PrEP persistence, using a log-rank test. Complete case analysis was used, assuming outcome data were missing completely at random. Analyses were done using Linux SAS version 9.4 (SAS/STAT 14.2, Cary, North Carolina, USA) and R version 3.6.2 (R Core Team, Vienna, Austria) with *survival* version 3.1.8 and *survminer* version 0.4.6 packages.

## Results

### Participant characteristics at enrollment

Of 646 women screened, 451 were eligible and enrolled, of whom 148 were enrolled from Harare, 141 from Cape Town, and 162 from Johannesburg. Of the 451 women enrolled, 427 (95%) initiated PrEP (412 at enrollment and 15 during follow-up); 212 were randomized to the standard adherence arm and 215 to the enhanced adherence arm (Fig 2). Retention at month 12 was 86% and did not differ by arm. The median age of women who started PrEP was 21 years (interquartile range [IQR] 19, 22), of whom 87% had completed some or all of secondary school, and most (90%) had a regular place to stay and store possessions (Table 1). The median number of sex partners in the past 3 months was 1 (IQR 1, 2), and 84% reported a primary sex partner, almost half of whom reported a primary partner who was 5 years older or more. Most (80%) thought their primary partner had other sex partners or were not sure. One quarter reported transactional sex in the month prior to enrollment. Women reported a median of 4 vaginal sex acts in the prior month, and 22% reported ≥1 anal sex acts in the past month. A minority (20%) reported always using condoms with vaginal sex and anal sex, one-third reported never or rarely using condoms with vaginal sex, and one quarter reported never using condoms with anal sex. Intimate partner violence in the prior year was reported by 50%, and two-thirds reported symptoms of trauma. Mild to moderate depression symptoms were reported by 61% of women, based on a Center for Epidemiologic Studies-Depression (CES-D) score ≥10 [20,21]. Long-acting contraceptives (i.e., injectables, implants, or intrauterine devices) were used by 68%. Curable sexually transmitted infection (STI) prevalence was high: 29% *C*. *trachomatis*, 8% *N*. *gonorrhoeae*, 7% *T*. *vaginalis*, and <1% syphilis seropositivity. The median VOICE risk score was 7, and 16% reported a moderate or high chance of acquiring HIV in the next year. Women reported positive attitudes about PrEP and low anticipated PrEP stigma, and 82% planned to disclose their PrEP use.

**Table 1:** Baseline demographic and behavioral characteristics of women who initiated PrEP in HPTN 082.

Baseline characteristic	Standard adherence support (*N* = 212)	Enhanced adherence support (*N* = 215)	Total (*N* = 427)
**Age (years)**			
Median (IQR)	21 (19, 23)	21 (19, 22)	21 (19, 22)
**Education**			
Primary school	4 (2%)	5 (2%)	9 (2%)
Secondary school	184 (87%)	187 (87%)	371 (87%)
College or university	24 (11%)	23(11%)	47 (11%)
Ever dropped out of school	67 (32%)	58 (27%)	125 (29%)
**Number of sex partners in past 3 months (*N* = 372)**			
Median (IQR)	1 (1, 2)	1 (1, 2)	1 (1, 2)
**Primary sex partner in the past 3 months (*N* = 356)**	174 (83%)	182 (85%)	356 (84%)
**Age difference with primary partner (*N* = 329)**			
Partner is same age or younger	6 (4%)	20 (12%)	26 (8%)
Partner is 1–5 years older	85 (52%)	74 (45%)	159 (48%)
Partner is >5 years older	72 (44%)	72 (43%)	144 (44%)
**HIV status of primary partner (*N* = 284)**			
HIV negative	112 (79%)	97 (68%)	209 (74%)
HIV positive	1 (1%)	2 (1%)	3 (1%)
Does not know	27 (19%)	42 (30%)	69 (25%)
**Thinks partner has other partners (*N* = 356)**			
Yes	54 (31%)	62 (34%)	116 (33%)
No	44 (25%)	25 (14%)	69 (19%)
Does not know	74 (43%)	94 (52%)	168 (47%)
**Vaginal sex acts, past month (*N* = 338)**			
Median (IQR)	4 (2, 8)	4 (2, 8)	4 (2, 8)
**Condom use with vaginal sex, past month (*N* = 331)**			
Always	40 (24%)	28 (17%)	68 (21%)
Often	20 (12%)	18 (11%)	38 (11%)
Sometimes	49 (30%)	57 (34%)	106 (32%)
Rarely	28 (17%)	24 (14%)	52 (16%)
Never	26 (16%)	39 (23%)	65 (20%)
**Anal sex acts, past month (*N* = 296)**			
None	117 (79%)	115 (78%)	232 (78%)
1–2	10 (7%)	7 (5%)	17 (6%)
≥2	22 (15%)	25 (17%)	47 (16%)
Any transactional sex in the past month^*^	51 (24%)	46 (21%)	97 (23%)
VOICE risk score^***^ Median (IQR)	7 (6, 8)	7 (6, 8)	7 (6, 8)
**CES-D depression score** ≥10^****^ **(*N* = 418)**	126 (59%)	133 (62%)	259 (61%)
**Any intimate partner violence, past year (*N* = 425)**	100 (48%)	116 (54%)	216 (51%)
Physical	40 (19%)	48 (22%)	88 (20%)
Emotional	74 (35%)	89 (41%)	163 (38%)
Forced sexual contact	19 (9%)	21 (10%)	40 (9%)
Felt unsafe or in danger	38 (18%)	48 (22%)	86 (20%)
Trauma symptoms^**^ **(*N* = 425)**	137 (65%)	152 (71%)	289 (68%)
**Contraceptive use**			
Intrauterine device	3 (1%)	1 (1%)	4 (1%)
Injectable	92 (43%)	92 (43%)	184 (43%)
Implant	55 (26%)	46 (21%)	101 (24%)
Oral	24 (11%)	29 (13%)	53 (12%)
Condoms only	28 (13%)	36 (17%)	64 (15%)
Sterilization	1 (<1%)	0 (0%)	1 (<1%)
None	9 (4%)	9 (4%)	18 (4%)
**Perceived risk of pregnancy, next year (*N* = 425)**			
Moderate or high	38 (19%)	42 (19%)	80 (18%)
**Perceived risk of HIV, next year (*N* = 425)**			
Moderate or high	36 (18%)	33 (15%)	69 (16%)
**Curable STI** Gonorrhea, chlamydia, trichomonas, and/or syphilis	79 (37%)	84 (39%)	163 (38%)
**PrEP stigma (agree or strongly agree) (*N* = 425)**			
Ashamed of using PrEP	10 (4%)	10 (5%)	20 (5%)
Embarrassed about using PrEP	13 (6%)	8 (3%)	21 (5%)
People will give her a hard time if they know she is on PrEP	34 (16%)	26 (12%)	60 (14%)
I am not following the rules of my community if I take PrEP	16 (8%)	19 (9%)	35 (8%)
I am at greater risk for physical violence if I take PrEP	25 (12%)	15 (7%)	40 (9%)
**Plan to disclose PrEP use (*N* = 425)**	348 (82%)	174 (83%)	174 (81%)

*Transactional sex is defined as having sex with a man because he provided her with or she expected he would provide her with food, clothes, cosmetics, items for children, transportation, cash, school fees, mobile phone air time, and other items.

^**^Maximum score of 10 on VOICE risk score (age ≤25, married or living with partner, partner provides her with financial and/or material support, primary partner has other partners, and alcohol use in the past 3 months). A minimum of 5 on the VOICE risk score was required for study eligibility.

^***^CES-D scale, maximum score of 30; CES-D ≥10 associated with mild depression. Missing values were imputed.

****Post-traumatic symptom scale including reports nightmares, intrusive thoughts, on guard or easily startled, or detached from activities or surroundings.

CES-D, Center for Epidemiologic Studies-Depression; HPTN, HIV Prevention Trials Network; IQR, interquartile range; PrEP, pre-exposure prophylaxis; STI, sexually transmitted infection.

### PrEP persistence

**Fig 3:**
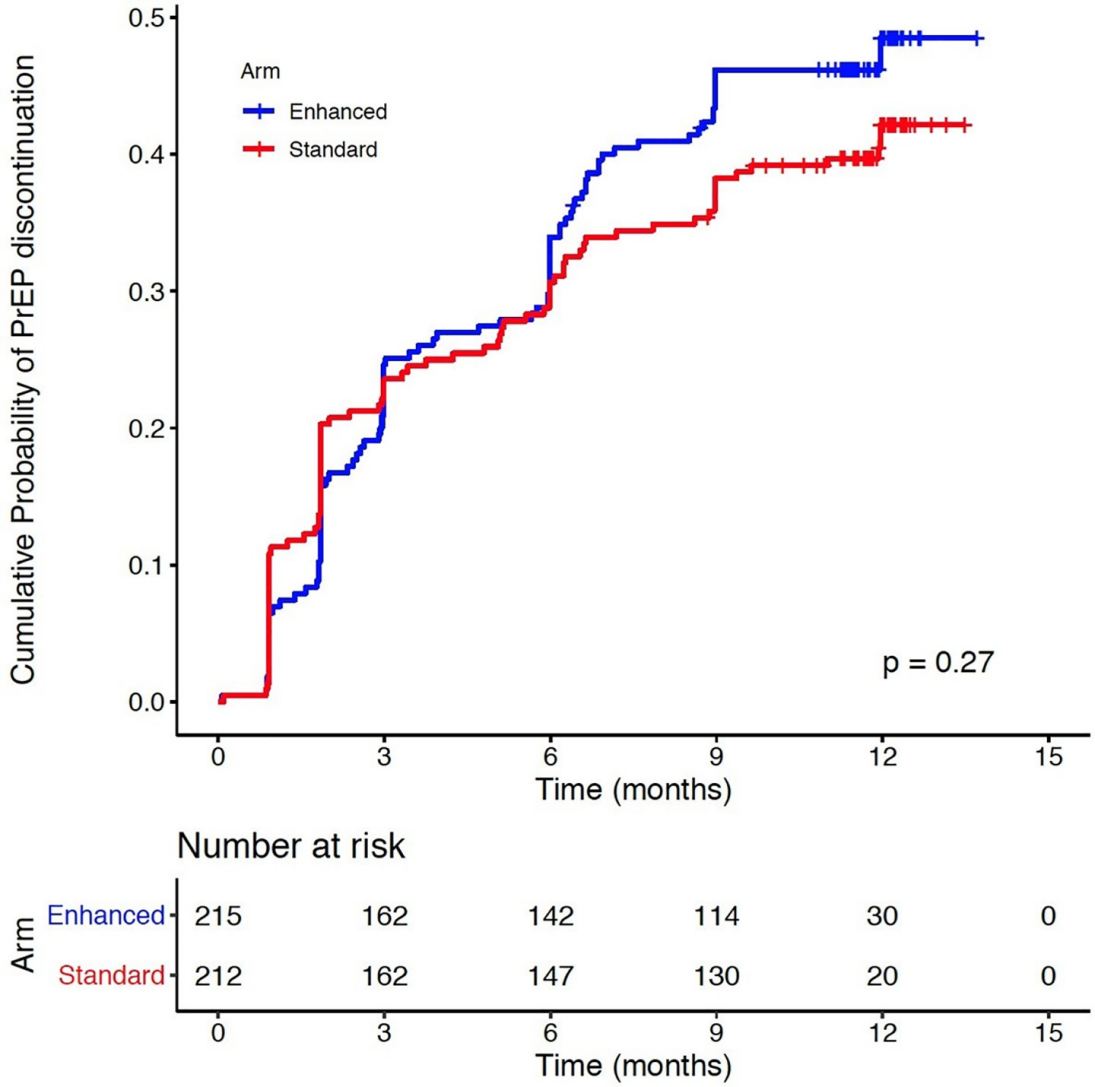
The Kaplan–Meier curve depicts time to first PrEP discontinuation due to (1) clinician- and participant-initiated PrEP discontinuations ≥30 days; or (2) a missed scheduled visit (assuming that all pills were used by the date of first missed scheduled study visit). Participants were censored at the time of HIV seroconversion or at the date of the last study visit. PrEP, pre-exposure prophylaxis.

At 12 months, 55% of women had not had a PrEP discontinuation, which did not differ by arm (Fig 3; *p* = 0.27).

A total of 114 holds occurred among 110 women, of which 40 were temporary, and 74 were permanent. Of these 74 permanent PrEP discontinuations, 22 discontinued on or before month 3, 23 on or before month 6, and 29 after month 6. The majority of temporary PrEP holds were for protocol-specified clinical reasons, including 24 participants with a ≥10% decrease in creatinine clearance from baseline, none of which declined below 60 ml/min. Clinical reasons for permanent discontinuation included pregnancy (*N* = 13), evaluation of possible HIV seroconversion (*N* = 8), side effects (*N* = 6), and decreased creatinine clearance (*N* = 3). Participants’ reasons to permanently discontinue PrEP were participant preference (*N* = 20), reporting they were no longer sexually active (*N* = 5), difficulty taking the pill (*N* = 5), social impact (*N* = 2), and other reasons (*N* = 10).

### Standard of care adherence intervention uptake

The SOC interventions were well accepted by participants in both arms. For the first 3 months of weekly 2-way SMS messages, 32% to 41% of outgoing messages were responded to by participants (the range representing differences by site), of whom 31% to 41% reported a problem (often due to scheduling appointments or questions), and only 1% requested help. Excluding participants with missing data about participation in the adherence clubs (37 overall, 17 in the standard, and 20 in the enhanced adherence arm), 50% attended 3 or more clubs in the first 6 months, 15% attended 2 clubs, 11% attended one, and 24% attended none, with similar proportions by arm.

### PrEP adherence and the effect of drug level feedback on adherence

Most (84%) of the 371 women with DBS samples from month 3 had detectable levels of TFV-DP, which decreased to 57% at month 6 and 31% at month 12 (Table 2). The median TFV-DP levels at months 3, 6, and 12 were 331 fmol/punch (IQR 80, 696), 66 fmol/punch (IQR 8, 614), and 8 fmol/punch (IQR 8, 83). Across both arms, 25% had high adherence at month 3, 21% at month 6, and 9% at month 12. Significant site differences were observed; at month 6, 28% had high adherence in the Johannesburg site, 25% in the Harare site, and 11% in the Cape Town site. High adherence tended to be persistent for an individual: Three quarters of those with high adherence at month 3 also had high adherence at month 6.

**Table 2:** PrEP adherence over 12 months by arm and overall, based on TFV-DP concentrations in DBS at months 3, 6, and 12 months, by intervention arm.

TFV-DP in DBS	3 months			6 months			12 months		
	**Standard (*N* = 186)**	**Enhanced (*N* = 185)**	**Overall (*N* = 371)**		**Standard (*N* = 186)**	**Enhanced (*N* = 185)**	**Overall (*N* = 371)**		**Standard (*N* = 186)**
**Detectable TFV-DP (>16.6 fmol/punch)**	152 (82%)	158 (85%)	310 (84%)	**Detectable TFV-DP (>16.6 fmol/punch)**	152 (82%)	158 (85%)	310 (84%)	**Detectable TFV-DP (>16.6 fmol/punch)**	152 (82%)
≥**700 fmol/punch**, among those with detectable TFV-DP	44 (24%)	48 (26%)	92 (25%)	≥**700 fmol/punch**, among those with detectable TFV-DP	44 (24%)	48 (26%)	92 (25%)	≥**700 fmol/punch**, among those with detectable TFV-DP	44 (24%)
**Median TFV-DP fmol/punch (IQR**), among all participants*	331 (80, 696)	330 (55, 661)	332 (93, 703)	**Median TFV-DP fmol/punch (IQR**), among all participants	331 (80, 696)	330 (55, 661)	332 (93, 703)	**Median TFV-DP fmol/punch (IQR**), among all participants	331 (80, 696)

*The median TFV DP levels for participants with *detectable* TFV-DP were 485 fmol/punch at month 3, 539 fmol/punch at 6 month 6, and 305 fmol/punch at month 12, with no significant difference by arm.

DBS, dried blood spots; IQR, interquartile range; PrEP, pre-exposure prophylaxis; TFV-DP, tenofovir-diphosphate.

At month 6, 36 (20.1%) of 179 participants in the enhanced adherence support arm had high adherence, compared to 40 (21.7%) of 184 in the standard adherence support arm, for an adjusted odds ratio (OR) of 0.92 (95% confidence interval [CI] 0.55, 1.34, *p* = 0.76, Table 3). The proportion with high adherence was 1.3% lower in the enhanced adherence support arm (95% CI −9.5%, 6.9%). At months 2 and 3, 56 of women in the enhanced adherence support arm did not receive drug level counseling at a visit due to the results not being available at their visit or receiving incorrect counseling due to site transcription errors and were removed from the per-protocol analysis (Fig 2). The per-protocol analysis of the month 6 outcome (Table 3) included 290 participants; high adherence was observed in 17 (15.2%) of 112 in the enhanced arm and 40 (22.5%) of 178 in the standard arm, for an adjusted OR of 0.64 (95% CI 0.34, 1.21, *p* = 0.17). The proportion with high adherence was 6.5% lower in the enhanced adherence support arm (95% CI −15.8, 2.8%). A total of 56 women were missing DBS data at month 6 because they were lost to follow-up (32 in enhanced and 24 in standard arm); in a sensitivity analysis, if all were considered to have undetectable TFV-DP because they no longer had access to PrEP, the between arm findings on adherence remain similar.

**Table 3:** Analysis of DBS TFV-DP concentration (≥700 vs. <700 fmol/punch) at month 6 among PrEP initiators by adherence support arm.

Comparison	Standard adherence support arm	Enhanced adherence support arm	Adjusted OR (95% CI)	*P* value
**Intention-to-treat analysis**				
** TFV-DP ≥700 fmol/punch**	40/184 (21.7%)	36/179 (20.1%)	0.92 (0.55, 1.34)	0.76
Per-protocol analysis^*^				
** TFV-DP ≥700 fmol/punch**	40/178 (22.4%)	17/112 (15.2%)	0.64 (0.34, 1.21)	0.17

*The per-protocol analysis excluded women after they discontinued PrEP due to a clinical or laboratory hold and visits in the enhanced arm where women did not fully receive the adherence feedback (either because DBS results were not returned before their next visit or because drug level counseling did not correspond to the DBS drug levels from the laboratory).

CI, confidence interval; DBS, dried blood spots; OR, odds ratio; PrEP, pre-exposure prophylaxis; TFV-DP, tenofovir-diphosphate.

### HIV incidence, PrEP adherence, and viral resistance in seroconverters

Four women became infected over 386.8 person-years of follow-up for an HIV incidence of 1.0/100 person-years (95% CI 0.3, 2.6). All 4 seroconverters were in the standard adherence arm. For the seroconverter at month 3, her TFV-DP level was 0 fmol/punch at month 3; for the seroconverter at month 6, her TFV-DP levels were 559 fmol/punch at month 3 and 243 fmol/punch at month 6; and for the 2 seroconverters at month 12, the closest DBS before seroconversion was at month 6 with TFV-DP values of 74 and 0 fmol/punch. Three seroconverters had no resistance mutations; 1 had D67N (an NRTI mutation) and 4 NNRTI mutations (K101E, K103N, E138A, and G109A), which are unrelated to tenofovir or emtricitabine.

## Discussion

This open-label study of PrEP uptake, adherence, and persistence enrolled young South African and Zimbabwean women with multiple risks for which PrEP was indicated, including older partners, uncertainty about their partner’s HIV status and whether their partner had other sexual partners, high rates of intimate partner violence and depression symptoms, low rates of consistent condom use, and high prevalence of bacterial STIs. Encouragingly, interest in PrEP was high, and over half had uninterrupted PrEP refills through 12 months. The majority were using PrEP in the first 6 months based on detectable drug levels, although only one-fifth achieved high levels of adherence over the first 6 months. Drug level feedback did not improve adherence at month 6 compared to the SOC adherence support package.

The drug level feedback used a threshold of 500 fmol/punch at week 4 as DBS TFV-DP levels do not reach steady state until between weeks 4 and 6, and 700 fmol/punch at week 8 for the high adherence category, based on the DBS threshold reflecting an average of 4 pills per week which was associated with 100% effectiveness of PrEP among MSM [22]. A similar threshold of pill taking for 100% effectiveness among women has not been established, and there is some evidence that a threshold of 700 fmol/punch may underestimate PrEP adherence in African populations compared to pill taking based on electronic pill openings [23]. In the enhanced adherence support arm, drug level feedback about women’s PrEP use, based on categories of good, intermediate, or no use, was designed to be motivational (“more is better” for HIV protection) and to foster open communication and problem-solving about PrEP adherence challenges. The logistics of drug levels involved quick turnaround of shipping and testing in order to provide drug level counseling at the next monthly visit and was operationally challenging; for a substantial minority of drug-level counseling visits, either the results were not available or incorrect counseling was given due to transcription errors. Two different thresholds used to define the high adherence category at month 1 (≥500 fmol/punch) and month 2 (≥700 fmol/punch) could have contributed to errors in transcribing absolute values from the laboratory report into counseling categories at months 2 and 3. However, in the per-protocol analysis which excluded women with PrEP holds and errors in drug feedback counseling, there was no significant difference by arm in adherence levels at month 6.

Drug level feedback may not have improved adherence in this study because it was lagged by over a month, reflecting adherence in the 4 to 6 weeks prior to the visit when the DBS sample was obtained with counseling provided a month later. It may have been difficult for women to remember their pill-taking behavior and adherence barriers from approximately 2 months ago. In contrast, drug level feedback support among MSM in a US demonstration project found an improvement in adherence in 50% of the MSM who had undetectable plasma tenofovir levels, which indicates no PrEP use in the prior week [13]. The different impact of drug level feedback in these studies may be due to the shorter recall period about adherence behavior with the plasma tenofovir assay. Using intracellular TFV-DP based on DBS in this study, women may have doubted the drug levels if their memory about their PrEP use 2 months ago was not consistent with the category of counseling they received, or they may have been demotivated to use PrEP if they perceived that they were “failing” PrEP if they were told their adherence was less than a green category. Point-of-care urine tenofovir assays in development will be simpler to implement and should be evaluated to determine whether real-time adherence assessment facilitates adherence counseling and problem-solving and improves subsequent adherence [24,25].

The uptake of 2-way SMS, brief counseling, and the peer support adherence clubs may have been high enough that we could not detect an additional benefit of retrospective drug level feedback. Further analyses will evaluate the level of adherence at 6 months relative to the amount of exposure to the SOC adherence support interventions of weekly 2-way SMS, brief cognitive behavioral counseling, and the voluntary adherence peer support clubs.

At month 6, the majority of women had used PrEP, although only 21% had high adherence, and PrEP use steadily declined over the next 6 months of follow-up. Some of this was due to permanent or temporary discontinuations of PrEP due to conservative renal monitoring criteria, pregnancy, and participant choice. The protocol required holding PrEP for a 10% decline from baseline, even if the estimated creatine clearance was in the normal range with a median creatinine clearance of 102 ml/min. It is important that renal monitoring is not a barrier to PrEP implementation due to repeat visits for PrEP users or causing concerns about PrEP safety. Similarly, PrEP discontinuation for pregnancy was required in this protocol because guidelines about PrEP in pregnancy were under development at the time of this study. Experts [26] and WHO currently recommend PrEP during pregnancy, when women are at increased risk of HIV acquisition [27,28].

Declining PrEP use could be a result of increasing intervals between visits, from monthly visits to quarterly visits. This was also observed in other PrEP demonstration projects among young African women that used a similar visit schedule HPTN 067/ADAPT [29], Plus Pills [30], and young US MSM in the Adolescent Trials Network (ATN) 110/113 studies [31]. In an ongoing PrEP demonstration project among Kenyan and South African women, approximately 20% of women who discontinued PrEP in the first 3 months restarted PrEP within 6 months [32,33]. Strategies to maintain engagement and motivation for young people taking PrEP could include more frequent clinic visits, adherence clubs, or social media platforms such as WhatsApp chat groups. The monthly adherence clubs and WhatsApp chat groups were highly acceptable in this study and should be rigorously evaluated as PrEP support strategies for young African women.

Notably, in this cohort of young women with 39% prevalence of curable STIs, multiple risk factors for HIV, and moderate PrEP adherence, HIV incidence was only 1%. Other recent HIV prevention trials in South African women found HIV incidence of approximately 4% [1–3]. These other trials enrolled participants from the same sites, and, during the same time, and reported similar sexual behavior and STI prevalence, indicating that the HPTN 082 cohort is representative of AGYW in these communities at risk of HIV and that our findings are generalizable. All 4 of the breakthrough HIV infections in this study were among women with low or no use of PrEP prior to HIV seroconversion, similar to studies in MSM. To understand to what extent this low HIV incidence can be attributed to PrEP, additional research is needed to determine whether PrEP use and adherence was aligned with periods of higher risk resulting in “prevention-effective adherence” [34]. The low observed HIV incidence suggests that women benefited from the prevention services delivered in this study, which, in addition to PrEP, included youth-friendly clinics, STI diagnosis and treatment, contraceptive counseling and services, peer support through adherence clubs, staff contact through 2-way text messages, and counseling.

Limitations of this study include that recruitment occurred before there were national guidelines for PrEP in South Africa and Zimbabwe. There was very little community awareness about PrEP at the time of this study, which may have impacted adherence and persistence as women faced challenges with partner and family misperceptions that they were HIV infected because they were taking a pill associated with HIV treatment. We did not have the power to detect a modest effect of retrospective drug level feedback on subsequent PrEP adherence. There was not a placebo control due to ethical concerns about withholding an effective intervention, so the contribution of PrEP to the low HIV incidence cannot be determined.

In summary, oral PrEP is a novel first-generation biomedical HIV prevention product, and demonstration projects provide important lessons about PrEP delivery, uptake, adherence, and persistence, which are informative for scale-up and implementation. In this cohort of young South African and Zimbabwean women who had multiple risks for HIV, PrEP initiation rates were high, one quarter achieved high adherence at 3 months, a majority persisted with PrEP for 6 months, and HIV incidence was low. Thus, oral PrEP was used well enough for most young women to obtain some benefit in spite of adherence and persistence challenges. To increase the proportion who benefit and achieve more durable HIV protection from PrEP, effective and scalable strategies are needed to increase adherence and persistence to daily PrEP among young African women as well as additional options, particularly longer-acting PrEP formulations, for young women for whom consistent pill taking is not feasible.

## Supporting information

S1 Text Protocol. (PDF)

S2 Text Counseling manual. (PDF)

S3 Text CONSORT diagram. CONSORT, CONsolidated Standards of Reporting Trials.
(PDF)

S4 Text Statistical analysis plan. (PDF)

## Abbreviations

AGYW: adolescent girls and young women
CES-D: Center for Epidemiologic Studies-Depression
CI: confidence interval
CONSORT: CONsolidated Standards of Reporting Trials
DBS: dried blood spots
HBV: hepatitis B virus
HPTN: HIV Prevention Trials Network
IQR: interquartile range
MSM: men who have sex with men
OR: odds ratio
PrEP: pre-exposure prophylaxis
SOC: standard of care
STI: sexually transmitted infection
TDF/FTC: tenofovir/emtricitabine
TFV-DP: tenofovir-diphosphate

## References

[1] ECHO Trial Consortium. HIV incidence among women using intramuscular depot medroxyprogesterone acetate, a copper intrauterine device, or a levonorgestrel implant for contraception: a randomised, multicentre, open-label trial. Lancet. 2019;394:303–13. doi: 10.1016/S0140-6736(19)31288-7 31204114 PMC6675739

[2] Baeten JM, Palanee-Phillips T, Brown ER, Schwartz K, Soto-Torres LE, Govender V, et al. Use of a Vaginal Ring Containing Dapivirine for HIV-1 Prevention in Women. N Engl J Med. 2016;(375):2121–32. doi: 10.1056/NEJMoa1506110 26900902 PMC4993693

[3] Nel A, van Niekerk N, Kapiga S, Bekker LG, Gama C, Gill K, et al. Safety and Efficacy of a Dapivirine Vaginal Ring for HIV Prevention in Women. N Engl J Med. 2016;375(22):2133–43. Epub 2016/12/14. doi: 10.1056/NEJMoa1602046 . 27959766

[4] Fonner VA, Dalglish SL, Kennedy CE, Baggaley R, O’Reilly KR, Koechlin FM, et al. Effectiveness and safety of oral HIV preexposure prophylaxis for all populations. AIDS. 2016;30(12):1973–83. Epub 2016/05/06. doi: 10.1097/QAD.0000000000001145 ; PubMed Central ID: 4949005. 27149090 PMC4949005

[5] Hanscom B, Janes HE, Guarino PD, Huang Y, Brown ER, Chen YQ, et al. Preventing HIV-1 Infection in Women using Oral Pre-Exposure Prophylaxis: A Meta-analysis of Current Evidence. J Acquir Immune Defic Syndr. 2016;73:606–8. Epub 2016/08/24. doi: 10.1097/QAI.0000000000001160 . 27846073 PMC5175411

[6] Van Damme L, Corneli A, Ahmed K, Agot K, Lombaard J, Kapiga S, et al. Preexposure prophylaxis for HIV infection among African women. N Engl J Med. 2012;367(5):411–22. Epub 2012/07/13. doi: 10.1056/NEJMoa1202614 . 22784040 PMC3687217

[7] Marrazzo JM, Ramjee G, Richardson BA, Gomez K, Mgodi N, Nair G, et al. Tenofovir-based preexposure prophylaxis for HIV infection among African women. N Engl J Med. 2015;372(6):509–18. doi: 10.1056/NEJMoa1402269 ; PubMed Central ID: 4341965. 25651245 PMC4341965

[8] Corneli AL, McKenna K, Perry B, Ahmed K, Agot K, Malamatsho F, et al. The Science of Being a Study Participant: FEM-PrEP Participants’ Explanations for Overreporting Adherence to the Study Pills and for the Whereabouts of Unused Pills. J Acquir Immune Defic Syndr. 2015;68(5):578–84. doi: 10.1097/QAI.0000000000000525 . 25761233

[9] van der Straten A, Stadler J, Luecke E, Laborde N, Hartmann M, Montgomery ET. Perspectives on use of oral and vaginal antiretrovirals for HIV prevention: the VOICE-C qualitative study in Johannesburg, South Africa. J Int AIDS Soc. 2014;17(3 Suppl 2):19146. Epub 2014/09/17. doi: 10.7448/IAS.17.3.19146 . 25224610 PMC4163995

[10] van der Straten A, Stadler J, Montgomery E, Hartmann M, Magazi B, Mathebula F, et al. Women’s experiences with oral and vaginal pre-exposure prophylaxis: the VOICE-C qualitative study in Johannesburg, South Africa. PLoS ONE. 2014;9(2):e89118. Epub 2014/03/04. doi: 10.1371/journal.pone.0089118 ; PubMed Central ID: 3931679. 24586534 PMC3931679

[11] Montgomery ET, Mensch B, Musara P, Hartmann M, Woeber K, Etima J, et al. Misreporting of Product Adherence in the MTN-003/VOICE Trial for HIV Prevention in Africa: Participants’ Explanations for Dishonesty. AIDS Behav. 2017;21(2):481–91. Epub 2016/11/20. doi: 10.1007/s10461-016-1609-1 ; PubMed Central ID: 5290166. 27858268 PMC5290166

[12] Seewoodharry MD, Maconachie GDE, Gillies CL, Gottlob I, McLean RJ. The Effects of Feedback on Adherence to Treatment: A Systematic Review and Meta-analysis of RCTs. Am J Prev Med. 2017;53(2):232–40. Epub 2017/05/01. doi: 10.1016/j.amepre.2017.03.005 . 28456347

[13] Landovitz RJ, Beymer M, Kofron R, Amico KR, Psaros C, Bushman L, et al. Plasma Tenofovir Levels to Support Adherence to TDF/FTC Preexposure Prophylaxis for HIV Prevention in MSM in Los Angeles, California. J Acquir Immune Defic Syndr. 2017;76(5):501–11. Epub 2017/09/14. doi: 10.1097/QAI.0000000000001538 ; PubMed Central ID: 5681370. 28902074 PMC5681370

[14] Drain PK, Dorward J, Violette LR, Quame-Amaglo J, Thomas KK, Samsunder N, et al. Point-of-care HIV viral load testing combined with task shifting to improve treatment outcomes (STREAM): findings from an open-label, non-inferiority, randomised controlled trial. Lancet HIV. 2020;7(4):e229–e37. Epub 2020/02/28. doi: 10.1016/S2352-3018(19)30402-3 ; PubMed Central ID: 7183312. 32105625 PMC7183312

[15] Fernandez MI, Hosek S, Warren JC, Jacobs RJ, Hernandez N, Martinez J. Development of an easy to use tool to assess HIV treatment readiness in adolescent clinical care settings. AIDS Care. 2011;23(11):1492–9. Epub 2011/10/26. doi: 10.1080/09540121.2011.565020 ; PubMed Central ID: 3203751. 22022853 PMC3203751

[16] Balkus J, Brown E, Palanee T, Nair G, Gafoor Z, Zhang J, et al. An Empiric HIV RIsk Scoring Tool to Predict HIV-1 Acquistion in African Women. J Acquir Immun Defic Syndr. 2016;72(3):333–4. doi: 10.1097/qai.0000000000000974 26918545 PMC4911322

[17] Anderson PL, Liu AY, Castillo-Mancilla JR, Gardner EM, Seifert SM, McHugh C, et al. Intracellular Tenofovir-Diphosphate and Emtricitabine-Triphosphate in Dried Blood Spots following Directly Observed Therapy. Antimicrob Agents Chemother. 2018;62(1). Epub 2017/10/19. doi: 10.1128/AAC.01710-17 ; PubMed Central ID: 5740314. 29038282 PMC5740314

[18] Castillo-Mancilla JR, Zheng JH, Rower JE, Meditz A, Gardner EM, Predhomme J, et al. Tenofovir, emtricitabine, and tenofovir diphosphate in dried blood spots for determining recent and cumulative drug exposure. AIDS Res Human Retroviruses. 2013;29(2):384–90. Epub 2012/09/01. doi: 10.1089/AID.2012.0089 ; PubMed Central ID: 3552442. 22935078 PMC3552442

[19] Schwartz GJ, Work DF. Measurement and estimation of GFR in children and adolescents. Clin J Am Soc Nephrol. 2009;4(11):1832–43. Epub 2009/10/13. doi: 10.2215/CJN.01640309 . 19820136

[20] Siddaway AP, Wood AM, Taylor PJ. The Center for Epidemiologic Studies-Depression (CES-D) scale measures a continuum from well-being to depression: Testing two key predictions of positive clinical psychology. J Affect Disord. 2017;213:180–6. Epub 2017/03/04. doi: 10.1016/j.jad.2017.02.015 ; PubMed Central ID: 6191531. 28254608 PMC6191531

[21] Baron EC, Davies T, Lund C. Validation of the 10-item Centre for Epidemiological Studies Depression Scale (CES-D-10) in Zulu, Xhosa and Afrikaans populations in South Africa. BMC Psychiatry. 2017;17(1):6. doi: 10.1186/s12888-016-1178-x 28068955 PMC5223549

[22] Grant RM, Anderson PL, McMahan V, Liu A, Amico KR, Mehrotra M, et al. Uptake of pre-exposure prophylaxis, sexual practices, and HIV incidence in men and transgender women who have sex with men: a cohort study. Lancet Infect Dis. 2014;14(9):820–9. Epub 2014/07/30. doi: 10.1016/S1473-3099(14)70847-3 . 25065857 PMC6107918

[23] Pyra M, Anderson P, Haberer JE, Heffron R, Celum C, Asiimwe S, et al. Tenofovir-Diphosphate as a Marker of HIV Pre-exposure Prophylaxis Use Among East African Men and Women. Front Pharmacol. 2019;10(401). doi: 10.3389/fphar.2019.00401 31057407 PMC6478885

[24] Gandhi M, Bacchetti P, Spinelli MA, Okochi H, Baeten JM, Siriprakaisil O, et al. Brief Report: Validation of a Urine Tenofovir Immunoassay for Adherence Monitoring to PrEP and ART and Establishing the Cutoff for a Point-of-Care Test. J Acquir Immune Defic Syndr. 2019;81(1):72–7. Epub 2019/01/22. doi: 10.1097/QAI.0000000000001971 ; PubMed Central ID: 6456396. 30664078 PMC6456396

[25] Lalley-Chareczko L, Clark D, Conyngham C, Zuppa A, Moorthy G, Mounzer K, et al. Delivery of TDF/FTC for Pre-exposure Prophylaxis to Prevent HIV-1 Acquisition in Young Adult Men Who Have Sex With Men and Transgender Women of Color Using a Urine Adherence Assay. J Acquir Immune Defic Syndr. 2018;79(2):173–8. Epub 2018/06/16. doi: 10.1097/QAI.0000000000001772 . 29905593

[26] Joseph Davey DL, Pintye J, Baeten JM, Aldrovandi G, Baggaley R, Bekker LG, et al. Emerging evidence from a systematic review of safety of pre-exposure prophylaxis for pregnant and postpartum women: where are we now and where are we heading? J Int AIDS Soc. 2020;23(1):e25426. Epub 2020/01/09. doi: 10.1002/jia2.25426 ; PubMed Central ID: 6948023. 31912985 PMC6948023

[27] Thomson KA, Hughes J, Baeten JM, John-Stewart G, Celum C, Cohen CR, et al. Increased Risk of HIV Acquisition Among Women Throughout Pregnancy and During the Postpartum Period: A Prospective Per-Coital-Act Analysis Among Women With HIV-Infected Partners. J Infect Dis. 2018;218(1):16–25. Epub 2018/03/08. doi: 10.1093/infdis/jiy113 ; PubMed Central ID: 5989601. 29514254 PMC5989601

[28] Graybill LA, Kasaro M, Freeborn K, Walker JS, Poole C, Powers KA, et al. Incident HIV among pregnant and breast-feeding women in sub-Saharan Africa: a systematic review and meta-analysis. AIDS. 2020;34(5):761–76. Epub 2020/03/14. doi: 10.1097/QAD.0000000000002487 ; PubMed Central ID: 7275092. 32167990 PMC7275092

[29] Bekker LG, Roux S, Sebastien E, Yola N, Amico KR, Hughes JP, et al. Daily and non-daily pre-exposure prophylaxis in African women (HPTN 067/ADAPT Cape Town Trial): a randomised, open-label, phase 2 trial. Lancet HIV. 2018;5(2):e68–e78. Epub 2017/10/08. doi: 10.1016/S2352-3018(17)30156-X ; PubMed Central ID: 6107917. 28986029 PMC6107917

[30] Gill K, Dietrich J, Gray G, Pidwell T, Kayamba F, Bennie T, et al. Pluspills: an open label, safety and feasibility study of oral pre-exposure prophylaxis (PrEP) in 15–19 year old adolescents in two sites in South Africa. 9th IAS Conference on HIV Science; July 23–26; Paris, France2017.

[31] Hosek SG, Rudy B, Landovitz R, Kapogiannis B, Siberry G, Rutledge B, et al. An HIV Preexposure Prophylaxis Demonstration Project and Safety Study for Young MSM. J Acquir Immune Defic Syndr. 2017;74(1):21–9. Epub 2016/09/16. doi: 10.1097/QAI.0000000000001179 ; PubMed Central ID: 5140725. 27632233 PMC5140725

[32] Rousseau-Jemwa E, Bekker L-G, Bukusi E, Delany-Moretlwe S, Omollo V, Traveill D, et al. Early persistence of HIV pre-exposure prophylaxis (PrEP) in African adolescent girls and young women (AGYW) from Kenya and South Africa. HIV Research for Prevention Meeting (HIVR4P); October 21–25; Madrid, Spain 2018.

[33] Omollo V, Odoyo J, Travill D, Rousseau-Jemwa L, Kidoguchi L, Bekker L, et al. PrEP re-initiation after interruption by adolescent girls and young women in Kenya and South Africa. 10th IAS Conference on HIV Science; July 21–24; Mexico City, Mexico2019.

[34] Haberer JE, Kidoguchi L, Heffron R, Mugo N, Bukusi E, Katabira E, et al. Alignment of adherence and risk for HIV acquisition in a demonstration project of pre-exposure prophylaxis among HIV serodiscordant couples in Kenya and Uganda: a prospective analysis of prevention-effective adherence. J Int AIDS Soc. 2017;20(1):21842. Epub 2017/07/26. doi: 10.7448/IAS.20.1.21842 ; PubMed Central ID: 5577705. 28741331 PMC5577705

