# Supplementary material for: PrEP uptake, persistence, adherence, and effect of retrospective drug level feedback on PrEP adherence among young women in southern Africa: Results from HPTN 082, a randomized controlled trial
Source: PLoS Med. 2021 Jun 18;18(6):e1003670. doi: 10.1371/journal.pmed.1003670 (PMC8253429; doi:10.1371/journal.pmed.1003670)
Supplement: S2 Text — (PDF) [file pmed.1003670.s002.pdf]

HPTN 082:
The HERS Study

ADHERENCE INTERVENTION COUNSELING MANUAL

Updated: 1 May 2017

SESSION OVERVIEW

Session	Topics	Activities
Session 1 (Enrollment)	PrEP information 101, PrEP adherence overview, social support, introduce AIM	1. Program Overview<br>2. PrEP 101<br>3. PrEP Adherence Overview<br>4. Introduce Problem Solving Barriers Using AIM<br>5. Social Support and PrEP Adherence
Session 2 (Week 4)	AIM Adherence review, exploring experience with PrEP, safe disclosure of PrEP to supportive others	1. Welcome Back & AIM check-in<br>2. Getting to Know your PrEP experience<br>3. Safe Talk- To disclose or not to disclose that I am on PrEP?
Session 3 (Week 8)	AIM adherence review, Gender dynamics, healthy sexuality, PrEP disclosure to sexual partners, reproductive decision making	1. Welcome back & AIM Check-in<br>2. Drug-Level Feedback<br>3. Being a Sexual Person<br>4. Reproductive health decision making & PrEP<br>5. Negotiating PrEP Use in a Sexual relationship
Session 4 (Week 13)	AIM adherence review, impact of emotions and stress on adherence, coping with stress	1. Welcome back & AIM Check-In<br>2. Drug-Level Feedback<br>3. Stress 101<br>4. Coping with Stress<br>5. Relaxation
Booster Session #1 (week 26)	AIM adherence review, begin discussion of future PrEP use	1. Welcome back & AIM Check-In<br>2. PrEP Decisional Balance
Booster Session #2 (week 39)	AIM adherence review, action plan for PrEP in future	1. Welcome back & AIM Check-In<br>2. PrEP Decisional Balance<br>3. Referrals and Plan<br>4. Farewell

<u>Notes for Facilitators</u>

- Participants enrolling in HPTN 082 will have a choice to take PrEP or not, and that choice continues throughout the course of the study. Those who initially agree to take PrEP at enrollment will follow the schedule outlined above. Those who decide to accept PrEP later in the study will also begin with Session 1 and follow the above schedule. However, those that accept late may not receive all of the counseling sessions before the study ends.

- Drug level feedback which occurs in Sessions 3 & 4 is only applicable to those randomized to that arm. Please make note of participants' assignment prior to those sessions.

SESSION 1: ENROLLMENT VISIT

Session 1	Activities	Time
1-A.	Program Overview & Expectations	5 minutes
1-B.	PrEP 101	5 minutes
1-C.	PrEP Adherence Overview	5 minutes
1-D.	Introduce AIM	10 minutes
1-E.	Social Support & Wrap-Up	5 minutes
	Total	30 minutes

ACTIVITY 1-A: Program Overview **TIME: 5 minutes**

**STATE**	*Welcome to our first counseling session for the* ***HERS*** *Study (HPTN 082).* ***HERS*** *is designed to empower and teach young women skills they need to feel confident about using PrEP. There will be a counseling session at each study visit. Six sessions in total. The sessions are expected to take 20-30 minutes each.*<br><br>*I want to review some important guidelines with you about our approach to these sessions.*
**DISPLAY**	Expectations Worksheet
**DISCUSS**	What should you expect from me?<br><br>• Confidentiality unless you are in danger of harming yourself or others or of being harmed<br>• Collaboration with you during our sessions<br>• Focus on your health<br>• Respect for your ideas and opinions<br>• Open to feedback and willingness to adapt to your needs<br><br>What do I expect from you?<br><br>• Be on time and attend all sessions<br>• Prepared for each session<br>• Be open and honest<br>• Investment in staying well<br>• Willing to give feedback
**ALLOW**	time to share feedback on the expectations
**STATE**	*An important component of the intervention involves empowerment. Empowerment means your ability to make your own choices, to become stronger and more confident, especially in controlling your life and your rights. Can you give me some ways a woman's level of empowerment could affect how she lives her life?*
**ALLOW**	Time to discuss.
**STATE**	*Empowerment includes self-acceptance, responsibility for your own behaviors, and a willingness to make healthy sexual choices. Throughout these sessions, you will learn skills to help you with PrEP, such as problem solving and communication, and learn about resources available to assist you. You can use these skills throughout our sessions together but also throughout other aspects of your life. I will be here to assist you in this process and this is not something that you will be doing alone!*
**ASK**	*Are there any questions?*
**ALLOW**	Time to discuss

ACTIVITY 1-B: PrEP 101 **TIME: 5 MINUTES**

**STATE**	*Next we are going to focus on understanding what PrEP is and discussing any rumors you have heard about PrEP. I'm also going to ask you some questions to make sure we are on the same page when discussing PrEP. Don't worry if you don't know the answer. That is why we are here today. Remember knowledge helps us become more empowered.*
**ASK**	*First, what does PrEP stand for?*
**ALLOW**	Participant to respond.
**STATE**	*PrEP stands for "pre-exposure prophylaxis". PrEP is a way for people to protect themselves against HIV. It involves taking one pill a day. The pill contains two medicines that are also used to treat HIV. If you take PrEP and are exposed to HIV, these medicines can work to keep the virus from taking hold in your body.*
**ASK**	*How well does PrEP work?*
**ALLOW**	Participant to respond.
**STATE**	*When taken every day, PrEP can reduce the risk for HIV infection by more than 90%. However, PrEP may not work as well or at all if it is not taken consistently.*
**SHOW**	Visuals demonstrating PrEP (show Project Video)
**ASK**	*Let's review a few points from the video.*<br><br>*Is PrEP safe?*
**ALLOW**	Participant to respond.
**STATE**	*PrEP is very safe. A few people may experience some start-up symptoms such as upset stomach, but these are usually mild and go away within the first few weeks. Other side effects are very rare.*
**ASK**	*Why is it important to get tested regularly for HIV before and while taking PrEP?*
**ALLOW**	Participant to respond.
**STATE**	*Testing regularly is important to make sure that you are still HIV negative. If you become HIV positive, then you need to take full treatment.*
**ASK**	*Do you have to take PrEP forever?*
**ALLOW**	Participant to respond.
**STATE**	*PrEP is not lifelong. It is only for as long as you need it to help you stay HIV negative.*
**ASK**	*Do you have any other questions about PrEP in general?*

**ALLOW**	Time for discussion

Activity 1-C: PrEP Adherence Overview **TIME: 5 MINUTES**

**STATE**	*Now let's talk a little about adherence to PrEP. When I say adherence, I mean taking the PrEP pill as it is prescribed. In this case, perfect adherence would be taking the pill every single day. Studies have shown that PrEP works very well for preventing HIV if it is taken every day. PrEP may not work as well or at all when it is not taken daily.*
**ASK**	*What thoughts do you have about taking the PrEP pill every day?*<br>*What may get in the way of adhering to PrEP?*
**DISCUSS**	Some common barriers to taking PrEP on a daily basis. Consider some of following (but don't need to review them all):<br>• Travel<br>• Forgetting<br>• Possible PrEP side effects<br>• Partner/family conflicts<br>• School demands<br>• Stigma/privacy concerns<br>• Lack of social support<br><br>But also remind participant that many people don't report barriers to adherence at all.
**STATE**	*Now, that we have reviewed the importance of taking the PrEP medication daily, let's start talking about how you will take PrEP. Think about an average week in your life. Please fill out this calendar with the typical weekly schedule that you keep.*
**DISTRIBUTE**	"Planner for the Week" Handout with daily schedule
**ALLOW**	Time for participant to complete the handout
**STATE**<br><br><br><br><br>**ASK**	*Thank you filling out the planner. The idea of having you complete this planner is to get you to start thinking about things that you do on a daily basis. This way we can review your daily schedule for cues that may remind you when it is time to take your pill.*<br><br>*Is there anything that you do most every day that could serve as a cue or signal for you to take your pill?*
**ALLOW**	Time for participant to discuss their daily planner<br>If participant has trouble identifying daily activities, probe <u>if necessary</u>:<br>• Are there other activities that happen with some regularity throughout your day? These might be major activities (going to work and/or school, picking up a child from school), or minor activities (watching a certain TV show, listening to radio program, or brushing your teeth before bed).<br>• Does your typical weekend day differ from your typical week day? How?<br>• Do you ever stay overnight somewhere other than your home?

	• What might disrupt your schedule (e.g., kids staying home sick from school, socializing, travel)?
**IDENTIFY**	Cues for daily dosing
**WRITE**	Cue on daily planner.

ACTIVITY 1-D: Problem Solving Barriers Using AIM TIME: 10 MINUTES

**STATE**	*Now, I'd like for us to make an adherence plan. You and I just discussed things that might get in the way of you taking your PrEP as prescribed. Let's try to come up with a solution for each one. This will be your plan to follow, so that you don't miss any doses of your PrEP. We have talked about adherence being specific to taking your PrEP as prescribed. However, adherence also includes anticipating issues that may not be directly related to taking a pill but that can have a great influence on being able to take the pill. For example, if I miss my study appointment, I may not be able to get my PrEP medication refilled so I am not able to be adherent.*
**SHOW**	AIM handout
**INTRODUCE**<br><br>**STATE**	AIM Method<br><br>*Let's consider 3 steps you can do to improve your adherence and minimize barriers. It may take some work at first, but it can become automatic and part of your routine.*<br>1. **A:** Adherence goal – state it!<br>2. **I:** Identify problems with reaching the goal<br>3. **M:** Make a plan to overcome the problems and develop a back-up plan.<br>(Safren et al., 2008).
**ASK**	*Up until this point, we have only talked about your motivation for taking PrEP and sticking with a medication schedule. Now I would like to learn more about your own goals specific to PrEP adherence. Let's break down these goals so that we can properly help you be successful with taking PrEP.*
**HANDOUT**	"My Adherence Goals" worksheet
**STATE**	*First, let's set some goals for PrEP adherence. For example, a goal for getting appointments can be "I want to make it to all my scheduled appointments!"*
**ALLOW**	Participant to complete (A) section of the handout.
**REVIEW**	AIM worksheet goals with participant.<br><br>If participant has trouble setting goals, here are some sample probes for the (A) categories:<br>1. Getting to study visits

	- How often are your appointments? How do you plan to get to your appointments? Who reminds you to go? 2. Communicating with study team - Think about your next study visit. What questions do you want to ask about the medication? About side effects? About sexual behavior? 3. Coping with side effects - What side effects (if any) have you noticed? 4. Obtaining medication and health products - Where do you get your PrEP filled? How do you carry the PrEP? What happens if you run out of PrEP? Where can you get condoms? 5. Sticking with a daily medication schedule - When do you want to take your PrEP? How will you remember to take it? 6. Storing medication - Where will you keep you PrEP? If you leave home, will you take it with you? How will you carry your PrEP with you? 7. Reminder strategies - How do you usually remind yourself to take medication? What cues will help to remind you? 8. Handling slips and adherence - How would you feel if you forgot to take your PrEP one day? What would you do if you slept through a dose? What if you were traveling and forgot your PrEP? What if you got sick and didn't feel like taking it?
**STATE**	*This is a great starting point for us to think about adherence. Now, each time we meet we will review these goals on this worksheet. We will also complete the other sections of the worksheet by Identifying problems you've had reaching the goals and Making a plan to overcome those problems.*
**ASK**	*Do you have any questions about this activity?*
**ALLOW**	Time for discussion

Activity 1-E: Social Support **TIME: 5 MINUTES**

**STATE**	*Now, let's think about the relationships we have with people who support us in our lives. "Supportive others" are the people or groups of people who are most important in our lives. Supportive others may include parents, peers, family members, schools, youth groups, faith communities, and dating partners. We all rely on supportive others to listen when we need to talk, give us advice, and shape our ideas about the decisions we make and the consequences of each decision. Supportive people can help support us to take PrEPs and attend appointments as well. In this activity, let's identify the people who are supportive to you.*

**DISTRIBUTE**	"Who's On Your Matatu?" worksheet.
**HAVE**	Participant write her name on the line beneath the picture of the bus.
**ASK**	*If you could fill a bus or a matatu with the people in your life who are most important, who would be on that matatu?*
**STATE**	*Please create more seats if there are others you would like to invite on your matatu.*
**HAVE**	Participant fill in each "matatu seat" (represented by the oval) with a person/group that is most important in their lives such as family, friends, teachers, boyfriends/girlfriends, etc. Ask participants to include only people (i.e., no pets, inanimate objects, etc.).
**ALLOW**	5 minutes for participants to complete their buses.
**DISCUSS**	• *Who are the people/groups on your bus?*<br>• *Why are they important to you?*<br>• *Are there some that have more influence than others?*<br>• *Are these people/groups you can count on when you are in trouble or in need?*<br>• *Do they help you make good decisions? Always? Most of the time?*<br>• *Some times? Never?*<br>• *Do you feel good about the decisions they help you make?*
**STATE**	*Thank you for completing this activity. I'm glad to understand who is important to you. We will talk more about social support the next time that we meet. Thank you for time today. It was a pleasure meeting you and getting to know you. I look forward to spending more time with you over the course of the HERS study*
**REVIEW**	Next scheduled appointment time

Expectations

What should you expect from the therapist?

- Confidentiality unless you are in danger of harming yourself or others
- Collaboration with you during therapy
- Investment in keeping you well
- Respect for your ideas and opinions
- Open to feedback and willingness to adapt to your needs

What does the therapist expect from you?

- Be on time and attend all sessions
- Prepared for each session
- Open and honest with the therapist
- Investment in staying well
- Willing to give feedback

WHO'S ON YOUR MATATU?

Directions: Fill in each "matatu seat" (represented by the ovals) with each person/group who is most important in your life such as family, friends, teachers, boyfriends/girlfriends, etc. Please include only people (for example, no pets, inanimate objects, etc.). You can create as many "matatu seats" as needed.

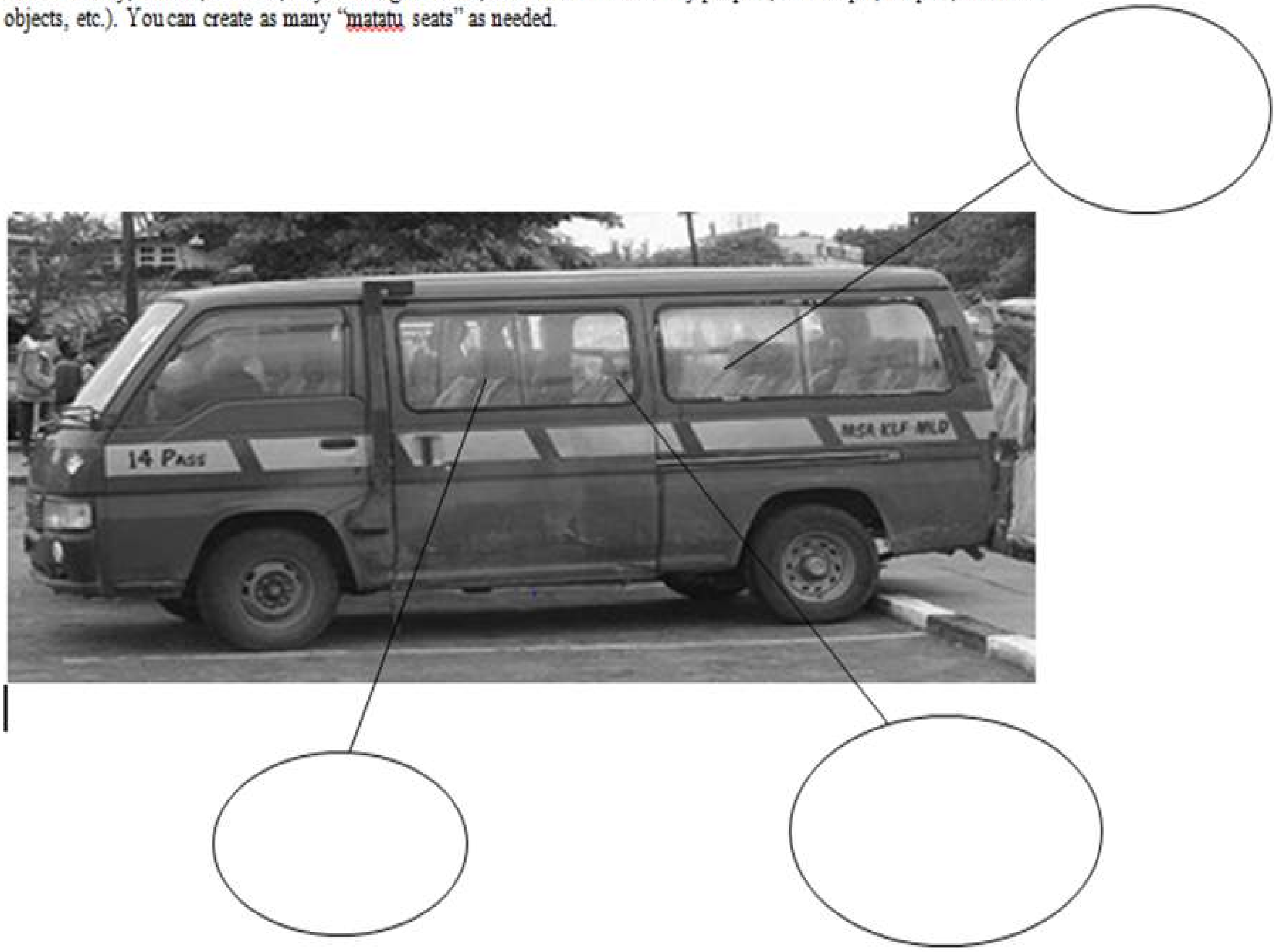

DAILY PLANNER WORKSHEET

Week of: ____________

Monday __________ **Tuesday** __________ **Wednesday** __________ **Thursday** __________

Friday __________ **Saturday** __________ **Sunday** __________ **Additional Notes**

www.timvandevall.com
Copyright © Dutch Renaissance Press LLC

MY ADHERENCE GOALS

A – Adherence Goal—State It!

I – Identify problems with reaching the goal as well as what you're already doing well

M – Make a plan to overcome the problems and develop a back-up plan

(A)

(I)

(M)

(A)

(I)

(M)

(A)

(I)

(M)

(A)

(I)

(M)

SESSION 2: WEEK 4 VISIT

Session 2	Activities	Time
2-A.	Welcome back and AIM Check-In	10 minutes
2-B.	Safe TALK, PrEP Disclosure	10 minutes
2-C.	Action Plan (optional) & Wrap-Up	5 minutes
	Total	25 minutes

ACTIVITY 2-A: Welcome Back & Check-In **TIME: 10 MINUTES**

<table>
<tr><td>STATE</td><td>Welcome back! I am glad you are here today. Today we will focus on learning more about adherence and problem solving. We will also talk about how to communicate that you are taking PrEP with other people. But first, I'd like to start by asking how your experience has been with PrEP thus far?</td></tr>
<tr><td>DISCUSS</td><td>Participant's experience

Possible probes:
Was taking PrEP harder or easier than you expected?
Are you feeling more or less motivated to take PrEP than last time?
Do you have any lingering concerns about continuing PrEP this month?</td></tr>
<tr><td>REVIEW</td><td>AIM goals with participant from last session</td></tr>
<tr><td>ASK</td><td>Last time, we introduced the concept of developing your PrEP adherence goals. Let's looking at your adherence goal worksheet from our last visit. How did you do?
Which areas of adherence do you feel like you do the best?
Which areas do you feel like you are not so good at?</td></tr>
<tr><td>REVIEW</td><td>Only the areas where the participant has the most trouble. You don't have to cover each area if it is already going well.

Potential goals and probes for Identifying Problems (I) and Making A Plan (M):
Getting to study visits
I: What causes you to miss appointments? What causes you to be late to appointments?
M: What will you do next time you miss an appointment? What will you do if you don't have transportation?
Communicating with study team
I: What causes you to not communicate with study team? (e.g., uncomfortable asking questions, visit feels rushed, etc.)
M: How might you practice communication with them? (e.g., write down questions, role play asking them)
Coping with side effects
I: Do side effects get in the way of you taking the PrEP? What have you tried to do for your side effects? Have you spoken to the study team?
M: Will you still be able to keep taking your PrEP as prescribed? What can you do to help manage side effects?
Obtaining medication and health products
I: What might cause you to run out of medicine? What might get in the way of you refilling the prescription?</td></tr>
</table>

	• M: How can you make sure you will not run out of medicine? What will help you remember?<br>5. Sticking with a daily medication schedule<br>• I: When do you seem to forget to take your PrEP? Do you take your PrEP at a time you regularly do something else? (e.g. breakfast, before bed, brushing teeth)<br>• M: What activities can you do at the same time as you take your PrEP to help you remember?<br>6. Storing medication<br>• I: Where do you keep your PrEP when you take it with you? Where do you keep it at home?<br>• M: Would you be able to use a pillbox or carry case?<br>7. Reminder strategies<br>• I: What would be helpful reminders to take your PrEP?<br>• M: Which reminder will work best for you?<br>8. Handling slips and adherence<br>• I: What thoughts do you think might keep you from getting back on track if you miss a dose/get off your routine? (e.g. look for all-or-nothing thinking, catastrophic thinking)<br>• M: What could you learn from a lapse that might help you avoid another one later on? What can you do to get back on track?
**REWORK**	A-I-M strategies if needed to address any barriers participant may have and make new goals as needed
**ASK**	*Is there anything else that might get in the way of your doing any of these steps?*
**ALLOW**	Time for discussion
**STATE**	*Taking pills every day can be challenging. Many people have problems taking their pills at some point. During any of our conversations, please feel comfortable telling me about pills you may have missed or taken late; I am asking because I want to make it easier for you to take them. We will continue to discuss this as we meet.*

ACTIVITY 2-B: SAFE TALK- HOW DO I DISCLOSE THAT I AM ON PREP?
TIME: 5 MINUTES

**STATE**	*The last time we met, we talked about some of the supportive people in your life – the people "on your matatu". Now let's talk about the possibility of telling a supportive person that you are taking PrEP. It is your choice to let someone whether you are taking PrEP. Often times, it may not possible to keep it from others.*<br><br>*How do you feel about telling people that you are taking PrEP?*
**DISCUSS**	Participants views on disclosing or not disclosing PrEP

**STATE**	*If you are struggling with how to tell someone that you are taking PrEP, here is an acronym, T.A.L.K., that can help guide you through the process.*
**HANDOUT**	"Safe TALK" handout.<br>**T**iming<br>**A**ssertive Communication<br>**L**ocation<br>**K**now What to Say
**STATE**	*Timing, Assertive Communication, Location, Know What to Say.*
**ASK**	**<u>Have the participant read text</u>** on the "SAFE Talk" handout.<br><br><u>TIMING</u><br>Choose an appropriate time to talk with your person. If the person that you need to talk with has a busy lifestyle, then it might be easier for you to set a meeting time. This way, each person's attention can be focused on the issue.<br><br><u>**A**SSERTIVE COMMUNICATION</u><br>Clearly tell the person how you feel and what you want or need by being honest and direct. Think carefully about your relationship and pay attention to others' responses. Depending on the specific person, you might have to address issues differently. Remember to use "I" statements, take deep breaths, keep a reasonable tone, and actively listen to the other person.<br><br><u>**L**OCATION</u><br>Choose a quiet place where you cannot be interrupted or overheard by others.<br><br><u>**K**NOWING WHAT TO SAY</u><br>Think about what you want to say in advance by sorting out your own feelings about the issue before talking with the other person. You might find that making a list or writing a letter of your thoughts and feelings will help you focus.
**DISCUSS**	Handout and answer any questions.
**EMPHASIZE**	*You have control over whether you tell people, who you tell and how you tell them. Think about what is best for you and make sure YOU are ready.*
**STATE**	*Now we are going to have you practice telling someone you are on PrEP by doing some role-playing, even if you aren't ready to tell someone yet. Choose someone who you may want to tell about PrEP in the future. Let me know who it is and provide me with some details about where the conversation is taking place. The more details you provide, the better. I will then pretend to be the person and react as I think the person would.*

**ALLOW**	Time for participant to prepare then Conduct the role-play.
**ASK**	*What was the most challenging thing about this role-play?*<br>*What part of this was easier than you thought it would be?*<br>*What surprised you going through this role-play?*
**ENCOURAGE**	Participant to share one thing they liked, and one thing they wish they would do differently.
**ALLOW**	Time for discussion

2-C: ACTION PLAN: DISCLOSURE (OPTIONAL) TIME: 5 MINUTES

**NOTE**	This activity is optional and ONLY for participants who are interested in telling someone about being on PrEP.
**STATE**	*You have said that you are interested in telling someone that you are taking PrEP. Let's develop an action plan to outline what steps you will take.*
**HANDOUT**	"Action Plan: Disclosure" handout
**STATE**	*Think about the specific person whom you would like to disclose your PrEP use to. Use this worksheet to think through the reasons why you want to disclose to that person. Then use this form to plan out the process. Bring together all of the elements of disclosure we talked about today. Decide when you would like to tell them, where you will have the talk, what you will say, and how you will do it. Finally, think about what the potential costs and benefits of disclosing to this person would be. Remember when thinking about costs and benefits, think primarily about how disclosing will affect you and your life.*
ALLOW	Participants 5 minutes to fill out their action plan. They may leave the worksheet with the counselor or take it home if they wish.
**STATE**	*Thank you for time today. It was a pleasure working with you again as part of the HERS study.*
**REVIEW**	Next scheduled appointment time

MY ADHERENCE GOALS

A – Adherence Goal—State It!

I – Identify problems with reaching the goal as well as what you're already doing well

M – Make a plan to overcome the problems and develop a back-up plan

(A) ____________________

(I) ____________________

(M) ____________________

(A) ____________________

(I) ____________________

(M) ____________________

(A) ____________________

(I) ____________________

(M) ____________________

(A) ____________________

(I) ____________________

(M) ____________________

SAFE T.A.L.K

TIMING

Choose an appropriate time to talk with your family or significant others. If the family member that you need to talk with has a busy lifestyle, then it might be easier for you to set a meeting time. This way, each person's attention can be focused on the issue.

ASSERTIVE COMMUNICATION

Clearly tell your family member or significant others how you feel and what you want or need by being honest and direct. Think carefully about your relationship and pay attention to others' responses. Depending on the specific person, you might have to address issues differently. Remember to use "I" statements, take deep breaths, keep a reasonable tone, and actively listen to your family member or significant others.

LOCATION

Choose a quiet place where you and your family member or significant others cannot be interrupted or overheard by others.

KNOWING WHAT TO SAY

Think about what you want to say in advance by sorting out your own feelings about the issue before talking with your family member or significant others. You might find that making a list or writing a letter of your thoughts and feelings will help you focus.

ACTION PLAN: DISCLOSURE

Think about one specific person to whom you would like to disclose your PrEP use. Let's use this worksheet to think through the reasons why you might want to disclose to that person. Then use this form to plan out the process.

*List all the reasons **WHY** you want to disclose to____________________.*

***WHO** am I disclosing to?*

***WHAT** will I say?*

***WHERE** will I say it?*

***WHEN** will I have this conversation?*

***HOW** will I do it?*

Potential Costs:

Potential Benefits:

SESSION 3: WEEK 8 VISIT

Activity	Time
**3A. Welcome Back & AIM Goals Check-In**	**10**
**3B. Healthy Sexuality**	**5**
**3C. Reproductive Decision Making**	**5**
**3D. Negotiating PrEP use with Sexual Partner**	**5**
**Total**	**25**

ACTIVITY 3-A. WELCOME BACK & CHECK-IN TIME: 5 MINUTES

**STATE**	*Welcome back! Last time, we reviewed your AIM worksheet to help you meet and sustain your PrEP adherence goals. Let's check in on these.*
**REVIEW**	AIM goals with participant
**ASK**	*In what areas of adherence have you been doing well? Are there areas where you are not doing so well?*
**REVIEW**	Only the areas where the participant has the most trouble. You don't have to cover each area if it is already going well.<br><br>Potential probes for Identifying Problems (I) and Making A Plan (M):<br>1. Getting to study visits<br>a. I: What causes you to miss appointments? What causes you to be late to appointments?<br>b. M: What will you do next time you miss an appointment? What will you do if you don't have transportation?<br>2. Communicating with study team<br>a. I: What causes you to not communicate with study team? (e.g., uncomfortable asking questions, visit feels rushed, etc.)<br>b. M: How might you practice communication with them? (e.g., write down questions, role play asking them)<br>3. Coping with side effects<br>a. I: Do side effects get in the way of you taking PrEP? What have you tried to do for your side effects? Have you spoken to the study team?<br>b. M: Will you still be able to keep taking your PrEP every day? What can you do to help manage side effects?<br>4. Obtaining medication and health products<br>a. I: What might cause you to run out of PrEP tablets? What might get in the way of you refilling the prescription?<br>b. M: How can you make sure you will not run out of PrEP tablets? What will help you remember?<br>5. Sticking with a daily medication schedule<br>a. I: When do you seem to forget to take your PrEP? Do you take your PrEP at a time you regularly do something else? (e.g. breakfast, before bed, brushing teeth)<br>b. M: What activities can you do at the same time as you take your PrEP to help you remember?<br>6. Storing medication<br>a. I: Where do you keep your PrEP tablets when you take it with you? Where do you keep it at home?<br>b. M: Would you be able to use a pillbox or carry case?<br>7. Reminder strategies<br>a. I: What would be helpful reminders to take your PrEP?<br>b. M: Which reminder will work best for you?<br>8. Handling slips and adherence

	a. I: What thoughts do you think might keep you from getting back on track if you miss a dose/get off your routine? (e.g. look for all-or-nothing thinking, catastrophic thinking)<br>b. M: What could you learn from a lapse that might help you avoid another one later on? What can you do to get back on track?
**CHECK**	Whether participant is randomized to receive drug level feedback or not. If not, skip this section and move on to next activity – Healthy Sexuality.
**STATE**	*Today, we have new information to share with you about your adherence and level of protection from HIV. This information comes from laboratory results that tell us how much of the PrEP drug was in your blood sample at your last study visit. These levels are an estimate of how well you took your pills in the few weeks prior to your last study visit.*
**SHOW**	Participant handout of 3 different "drug level" wireless signals
**ASK**	*These symbols are wireless signals, which we are using to represent the "strength" of your HIV protection based on your drug levels. Are you ready to hear about your PrEP drug levels?*
	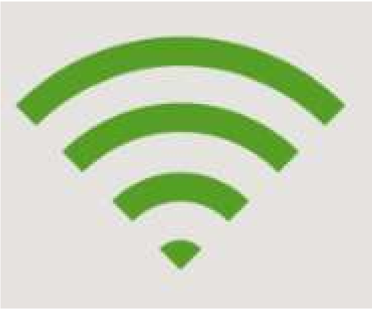<br>Key message: You are doing really well! Keep up the good work and remember that taking one PrEP pill every day is needed for strong protection against HIV.<br>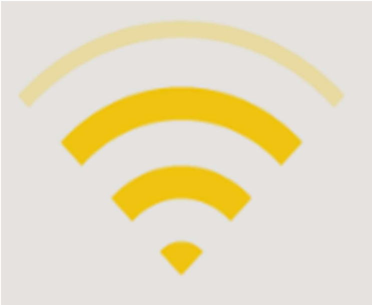<br>Key message: It looks like you are trying to take the PrEP pills, but may have missed some doses. Remember that taking one pill every day is needed for strong protection against HIV. How can we help you do even better?<br>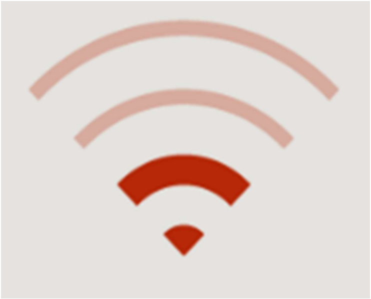<br>Key message: It looks like you haven't been able to take the PrEP pills. Is PrEP something that you are still interested in? If yes, how can we help you?
**ASK**	*How does this match up with what you expected to see? Do you have any questions about these results?*

**ALLOW**	Time for discussion; Provide encouragement for all

ACTIVITY 3-B: HEALTHY SEXUALITY **TIME: 5 MINUTES**

**STATE**	*Today, we will focus explore a different aspect of ourselves as it relates to PrEP adherence - Our sexuality. Sexuality can mean who you are attracted to, your sexual feelings about other people, the type of sex activities that you do, or even how you feel about sex.*
**ASK**	*In what ways do you think taking PrEP impacts your sexual activity?*
**ALLOW**	Participant to respond.
**STATE**<br><br><br><br><br>**ASK**	*Some of our opinions and beliefs about sex are influenced by our society, community, friends and family.*<br><br>*Let's spend some time talking about various views of sexuality among men and women.*<br><br>*1. How do you and your community view women and their sexuality?*<br>*a.. How do you feel (what do you think) about it?*<br>*2. How do you and your community view men and their sexuality?*<br>*a. How do you feel (what do you think) about it?*
**ALLOW**	Time for discussion
**STATE**	*Thank you for all your thoughtful answers. Healthy sexuality includes:*<br>• *Having the sexual knowledge and expertise to feel comfortable with yourself,*<br>• *Having the confidence to express yourself in your sexual relationships,*<br>• *Being comfortable with how you view your own sexuality and sexual behavior,*<br>• *Being knowledgeable and responsible regarding safer sex for you and your partner, and*<br>• *Being comfortable with your own body parts and body image*
**ALLOW**	Time for discussion

ACTIVITY 3-C: REPRODUCTIVE DECISION MAKING **TIME: 10 MINUTES**

**STATE**	*Another part of our sexuality is pregnancy and parenting.*
**ASK**	Probe:<br>*Are you currently a mother?*<br><br>*If yes, how many children do you plan to have?*

	If no, do you have plans to be a mother in the future?<br>*Have your desires to be a mother changed over time?*
**DISCUSS**	Participant's experience
**ASK**	*Ultimately, the decision to become pregnant and have a child is a very personal one. In order for you to weigh the pros and cons yourself, to decide if having a child is the right thing for YOU, you can use something called a decisional balance worksheet.*
**HANDOUT**	Decisional Balance: Having a Baby
**STATE**	*With a decisional balance sheet, you are listing pros and cons that are very specific to you, your life, your thoughts and your feelings. This allows you to weigh the drawbacks and benefits of all of your options in order to get a clearer understanding of what is right for you. Let's review this worksheet together*
**DISCUSS**	Decisional Balance Worksheet with participant. Help with pros and cons if needed.<br><br>Pros may include:<br>• Creating new life<br>• Having someone to love<br>• Having someone love you unconditionally<br>• Leaving behind a part of you when you die<br>• Not being alone<br><br>Cons may include<br>• Risk of infant death<br>• Monetary expense of having a child<br>• Risk of getting HIV from an HIV+ partner<br>• Stress<br>• Impact on school, career or lifestyle
**ASK**	*Let's say that you don't want to get pregnant or have more children right now. What are things you can do to prevent pregnancy?*
**ALLOW**	Participant to respond<br><br>Probe for current form of contraception
**ASK**	*Let's say you want to have a child or more children but you have a partner that is living with HIV. How can you do it safely?*
**ALLOW**	Participant to respond<br><br>Probe for PrEP as an option<br>Probe for HIV treatment for partner
**DISCUSS**	Any questions or concerns
**STATE**	*Thank you for being open in your discussion of sexuality today.*

ACTIVITY 3-D: Negotiating PrEP Use in a Sexual Relationship **TIME: 5 MINUTES**

**STATE**	*Part of healthy sexuality also means being open and honest with your sexual partners about your safer sex decisions.*
**STATE**	*You may decide that you want to talk to your husband, boyfriend or your partner about using PrEP at some point. This might seem a bit difficult, but if you prepare yourself, it will be easier. Remember last time with discussed the "Safe TALK" strategy?*<br><br>Show "Safe TALK" handout but review <u>only if necessary</u><br><br><u>**T**IMING</u><br>Choose an appropriate time to talk with your person. If the person that you need to talk with has a busy lifestyle, then it might be easier for you to set a meeting time. This way, each person's attention can be focused on the issue.<br><br><u>**A**SSERTIVE COMMUNICATION</u><br>Clearly tell the person how you feel and what you want or need by being honest and direct. Think carefully about your relationship and pay attention to others' responses. Depending on the specific person, you might have to address issues differently. Remember to use "I" statements, take deep breaths, keep a reasonable tone, and actively listen to the other person.<br><br><u>**L**OCATION</u><br>Choose a quiet place where you cannot be interrupted or overheard by others.<br><br><u>**K**NOWING WHAT TO SAY</u><br>Think about what you want to say in advance by sorting out your own feelings about the issue before talking with the other person. You might find that making a list or writing a letter of your thoughts and feelings will help you focus.<br><br>*Tell him some of the things you have learned about STIs and HIV. It's also important to negotiate and listen to him. Keep in mind that it's not only your right, but also your RESPONSIBILITY to make decisions that you will help you stay healthy.*<br><br>*It's very important to know what you will say in response to your partner's questions, complaints, or efforts to change your mind. You can anticipate his reactions and responses and make the conversation a little easier for you.*
**STATE**	*Let's practice discussing PrEP with your husband/boyfriend.*

**DISPLAY**	How to talk PrEP with your partner handout…<br><br>What if your partner says…<br><br>• I am faithful to you, you don't need PrEP<br>• PrEP is not effective.<br>• PrEP can make us sick.<br>• PrEP is not going to protect you from getting pregnant.
**ASK**	*How would you respond to these statements by your partner? Let's practice.*
**ROLE PLAY**	Different ways to respond to the partner statements
**DISCUSS**	Alternative responses with the participant.
**THANK**	Participant for sharing her feelings and being open and honest about the process of disclosure.

ACTIVITY 3-E: WRAP-UP **TIME: 5 MINUTES**

**STATE**	*Today we spent some time talking about PrEP and sexuality, family planning and communication with your sexual partner.*
**ASK**	*How might these topics help you this week?*
**DISCUSS**	Responses
**ASK**	*How could these strategies apply to your PrEP adherence?*
**ALLOW**	Time for discussion and reflection
**STATE**	*Thank you for your time today. It was a pleasure working with you again as part of the HERS study.*
**REVIEW**	Next scheduled appointment time

MY ADHERENCE GOALS

A – Adherence Goal—State It!

I – Identify problems with reaching the goal as well as what you're already doing well

M – Make a plan to overcome the problems and develop a back-up plan

(A)

(I)

(M)

(A)

(I)

(M)

(A)

(I)

(M)

(A)

(I)

(M)

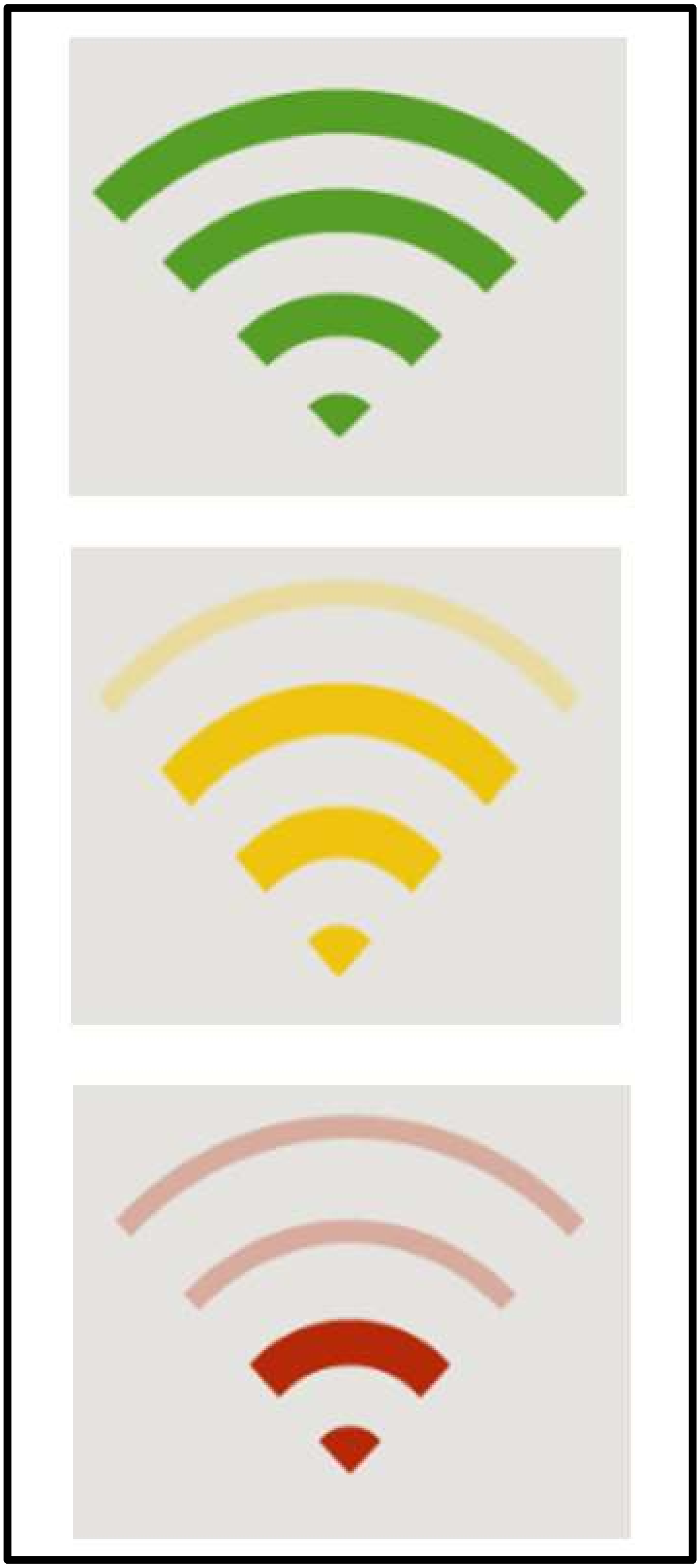

DECISIONAL BALANCE: HAVING A BABY

	BENEFITS/PROS	COSTS/CONS
Becoming Pregnant/ Having a Baby		
Not Becoming Pregnant/ Not Having a Baby		

How to talk PrEP with your partner…

Your partner says:

“I am faithful to you, you don’t need PrEP.”

“PrEP doesn’t work.”

“PrEP can make us sick.”

“PrEP is not going to protect you from getting pregnant.”

SESSION 4: WEEK 13 VISIT

**4-A: Welcome back & AIM Check-In**	**10 Minutes**
**4-B: Stress 101**	**5 minutes**
**4-C: Coping with Stress**	**5 minutes**
**4-D: Wrap-up**	**5 minutes**
**Total**	**25 minutes**

ACTIVITY 4-A: WELCOME BACK AND AIM CHECK-IN TIME: 5 MINUTES

**STATE**	*Welcome back! Last time, we reviewed your AIM worksheet to help you meet and sustain your PrEP adherence goals. Let's check in on these.*
**REVIEW**	AIM goals with participant
**ASK**	About any new or unresolved barriers
**REVIEW**	Only review areas where the participant has the most trouble.<br><br>Potential probes for Identifying Problems (I) and Making A Plan (M):<br>1. Getting to study visits<br>a. I: What causes you to miss appointments? What causes you to be late to appointments?<br>b. M: What will you do next time you miss an appointment? What will you do if you don't have transportation?<br>2. Communicating with study team<br>a. I: What causes you to not communicate with study team? (e.g., uncomfortable asking questions, visit feels rushed, etc.)<br>b. M: How might you practice communication with them? (e.g., write down questions, role play asking them)<br>3. Coping with side effects<br>a. I: Do side effects get in the way of you taking the PrEP? What have you tried to do for your side effects? Have you spoken to the study team?<br>b. M: Will you still be able to keep taking your PrEP as prescribed? What can you do to help manage side effects?<br>4. Obtaining medication and health products<br>a. I: What might cause you to run out of medicine? What might get in the way of you refilling the prescription?<br>b. M: How can you make sure you will not run out of medicine? What will help you remember?<br>5. Sticking with a daily medication schedule<br>a. I: When do you seem to forget to take your PrEP? Do you take your PrEP at a time you regularly do something else? (e.g. breakfast, before bed, brushing teeth)<br>b. M: What activities can you do at the same time as you take your PrEP to help you remember?<br>6. Storing medication<br>a. I: Where do you keep your PrEP when you take it with you? Where do you keep it at home?<br>b. M: Would you be able to use a pillbox or carry case?<br>7. Reminder strategies<br>a. I: What would be helpful reminders to take your PrEP?<br>b. M: Which reminder will work best for you?<br>8. Handling slips and adherence

	a. I: What thoughts do you think might keep you from getting back on track if you miss a dose/get off your routine? (e.g. look for all-or-nothing thinking, catastrophic thinking)<br>b. M: What could you learn from a lapse that might help you avoid another one later on? What can you do to get back on track?
**REWORK**	A-I-M strategies if needed to address any barriers participant may have
**REINFORCE**	Any progress and problem solving progress.
**CHECK**	Whether participant is randomized to receive drug level feedback or not. If not, skip this section and move on to next activity – Stress 101.
**STATE**	*We have new laboratory results that tell us how much of the PrEP drug was in your blood sample at your last study visit. Remember, these levels are an estimate of how well you took your pills in the few weeks prior to your last study visit.*
**SHOW**	Participant handout of 3 different "drug level" wireless signals
**ASK**	*Remember from our last session that these symbols are wireless signals, which we are using to represent the "strength" of your HIV protection based on your drug levels. Are you ready to hear about your PrEP drug levels?*

	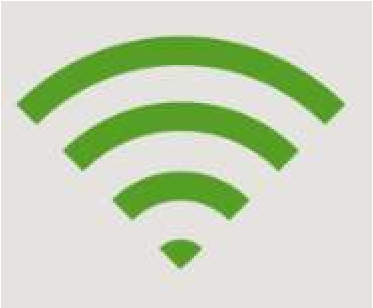<br>Key message: You are doing really well! Keep up the good work and remember that taking one PrEP pill every day is needed for strong protection against HIV.<br>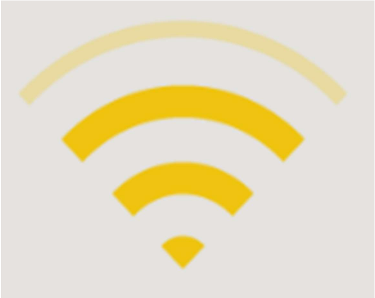<br>Key message: It looks like you are trying to take the PrEP pills, but may have missed some doses. Remember that taking one pill every day is needed for strong protection against HIV. How can we help you do even better?<br>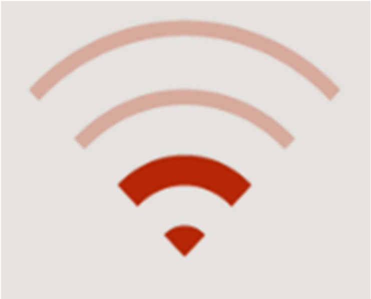<br>Key message: It looks like you haven't been able to take the PrEP pills. Is PrEP something that you are still interested in? If yes, how can we help you?
**ASK**	*How does this match up with what you expected to see? Do you have any questions about these results?*
**ALLOW**	Time for discussion; Provide encouragement for all

ACTIVITY 4-B: STRESS 101 **TIME: 5 MINUTES**

**STATE**	*We've been talking a lot these last few sessions about adherence and also disclosure of PrEP use. Sharing something personal about yourself with another person can be stressful. For the rest of the session today, we will talk about stress and emotions. We will also talk about ways to cope with the stressors of life.*
**ASK**	*What is stress?*
**ALLOW**	Participant to describe what stress means to her

**STATE**	*Stress is the EMOTIONAL and PHYSICAL reactions you experience because of changes and demands in your life. It is the feeling you get and the way your body reacts to these changes.*<br><br>*Stress can come from both POSITIVE and NEGATIVE situations. For example, we associate stress with events such as being sick, or worrying about your grades or your finances. But stress can also come from positive events, such as weddings, celebrating holidays, having a baby or starting a new relationship.*<br><br>*Stressors can also be MIXED – both positive and negative. A woman can be excited about having a new boyfriend, but she may be stressed about whether he is the right man for her.*
**HANDOUT**	**What Stresses You?** handout<br><br>What STRESSES You?<br>What are your 3 BIGGEST stressors?<br>1.____<br>2. ____<br>3. ____
**STATE**	*I would like you to take a few minutes to think of the things that cause you to feel stressed, then on the handout write your three biggest stressors.*
**ALLOW**	Time for participant to complete the handout.

ACTIVITY 4-C: COPING WITH STRESS **TIME: 5 MINUTES**

**STATE**	*Now we are going to focus on different ways to COPE or deal with stress. Coping can be described in many ways. For example, you might say you are "handling things", "taking care of business", "dealing with the situation", or "doing what you have to do".*<br><br>*Some methods of coping can be effective, and other methods of coping may not work so well. If you successfully cope with a situation, you improve your ability to handle the situation.*
**HANDOUT**	Relax Your Body and Relax Your Emotions handout
**REVIEW**	Ask **participant to read** the worksheet to you<br><br>**Relax Your Body**<br>**1. Deep Breathing**: While sitting, lying down or standing, close your eyes and breathe in slowly. Let your breath out for a count of 4 seconds. Take 10 of these super-relaxers any time you feel tense.

	**2. Stretching:** Practice simple stretches such as the neck stretch. Stretch your neck by gently rolling your head in a half circle, starting at one side, then dropping you r chin to your chest, then to the other side. Be careful not to roll your head back.<br><br>**3. Exercise:** All kinds of physical activity – walking, running, playing sports, dancing – help to reduce stress.<br><br>**4. Take a Break:** Ask family members to allow you at least 30 minutes of time alone.<br><br>**5. Eat Well:** Reduce caffeine (in coffee, tea, soda and chocolate) and alcohol intake. Take steps to eat healthy.<br><br>**Relax Your Emotions**<br>**1. Talk:** Take time to talk with a friend, partner or child. Express feelings you might have been holding in.<br><br>**2. Laugh:** Go see a funny movie, watch a funny video, or spend time with a friend who makes you laugh.<br><br>**3. Cry:** Crying can be as good a release as laughing. If you haven't cried in a long time, try listening to sad music or watching a sad movie.<br><br>**4. Read:** A good book is a great escape.<br><br>**5. Do Something You Love:** When you enjoy yourself, whether you are dancing, going to the park, or seeing friends, you relax your emotions.<br><br>**6. Write:** Another way to reduce stress is to write things down or to draw pictures about how you feel. When you are stressed or angry and have no one to talk to or are not comfortable with talking, try writing or drawing your feelings.
**STATE**	*Thanks so much for reading that. It's not always easy to identify coping strategies for stress. Sometimes it involves figuring out how you might change your behavior to reach the desired outcome.*<br><br>*To deal with stressful situations, we are going to apply something called the DECIDE model.*
**HANDOUT**	**DECIDE Model** handout
**ASK**	The <u>participant to read</u> the handout aloud.<br><br>**D = Define the problem.**<br>You might say "I need to figure out what is bothering me"<br>**E= Explain ways to change it.**<br>You might say "I need to think about what I can do about my problem"<br>**C= Choose the best option.**<br>You might say "I need to pick the best option for me"

	**I = It's time to act.**<br>You might say "I need to try out my choice"<br>**D= Did it work?**<br>You might say "Am I OK with the results?"<br>**E = Explore other solutions to my problem.**<br>You might say "If the results are not OK with me, I need to try another option"
**STATE**	*Now, let's look at the DECIDE model to determine how we can make choices and cope effectively with certain problems. Let's go back to your list of 3 biggest stressors. Pick one and we will apply the DECIDE model to it. I will check in with you during our next session to check on items "D = Did it Work?" and "E = explore other solutions".*
**ALLOW**	Time for participant to apply DECIDE steps to one of her stressors.
**STATE**	*Thank you for working on that stressor. DECIDE could also help us with stressors related to PrEP adherence.*<br><br>*Let's try it with the following situation.*<br>***A young woman has recently begun PrEP. She has been having a hard time remembering to take her medication at night. Her next scheduled appointment isn't for 3 months. She's not sure what to do and she's feeling very stressed about it.***<br><br>*Let's go through the model one letter at a time and see if we can think of examples of how to handle the situation.*<br><br>Have the participant go through each step - only use prompts when needed<br><br>**D = Define the problem.**<br>Prompt: I can't remember to take my PrEP pills every day<br>**E= Explain ways to change it.**<br>Prompt: I could stop taking the PrEP. I could go back to the clinic and talk to a nurse about it. I could talk to others that I know on PrEP.<br>**C= Choose the best option.**<br>Prompt: I will look at all my choices and pick the one that seems best for me.<br>**I = It's time to act.**<br>Prompt: I have decided that I need to call a nurse. I will work closely with them to make the best plan of action to help me continue to be healthy;<br>Or<br>I think I want to stop taking the PrEP, but I will talk to the nurse first.<br>**D= Did it work?**<br>Prompt: After speaking with the nurse, I developed a system to help remind me by leaving the pills next to my toothbrush.<br>**E = Explore other solutions to my problem.**<br>Prompt: This works for now, but if it doesn't work in the future I can go back to thinking about ways to change the situation and try something else.
**ALLOW**	Time for discussion

**STATE**	*Thank you for working with me today to discuss different approaches to handling life stress.*

ACTIVITY 4-D: REFLECTION & WRAP-UP TIME: 5 MINUTES

STATE	*For the past few sessions, we covered a lot of topics that relate to PrEP. We've talked about PrEP adherence, healthy sexuality for young women, and ways to identify and reduce stress in your life.*<br><br>*During our time together, I've noticed…*
**EXPRESS**	Personalized observations about participant's efforts and strengths.
**STATE**	*Thank you for your time today. It was a pleasure working with you again as part of the HERS study. Your study visits will be further apart now, but we will continue to meet briefly for each of your last 2 visits.*
**REVIEW**	Next scheduled appointment time

MY ADHERENCE GOALS

A – Adherence Goal—State It!

I – Identify problems with reaching the goal as well as what you're already doing well

M – Make a plan to overcome the problems and develop a back-up plan

(A)

(I)

(M)

(A)

(I)

(M)

(A)

(I)

(M)

(A)

(I)

(M)

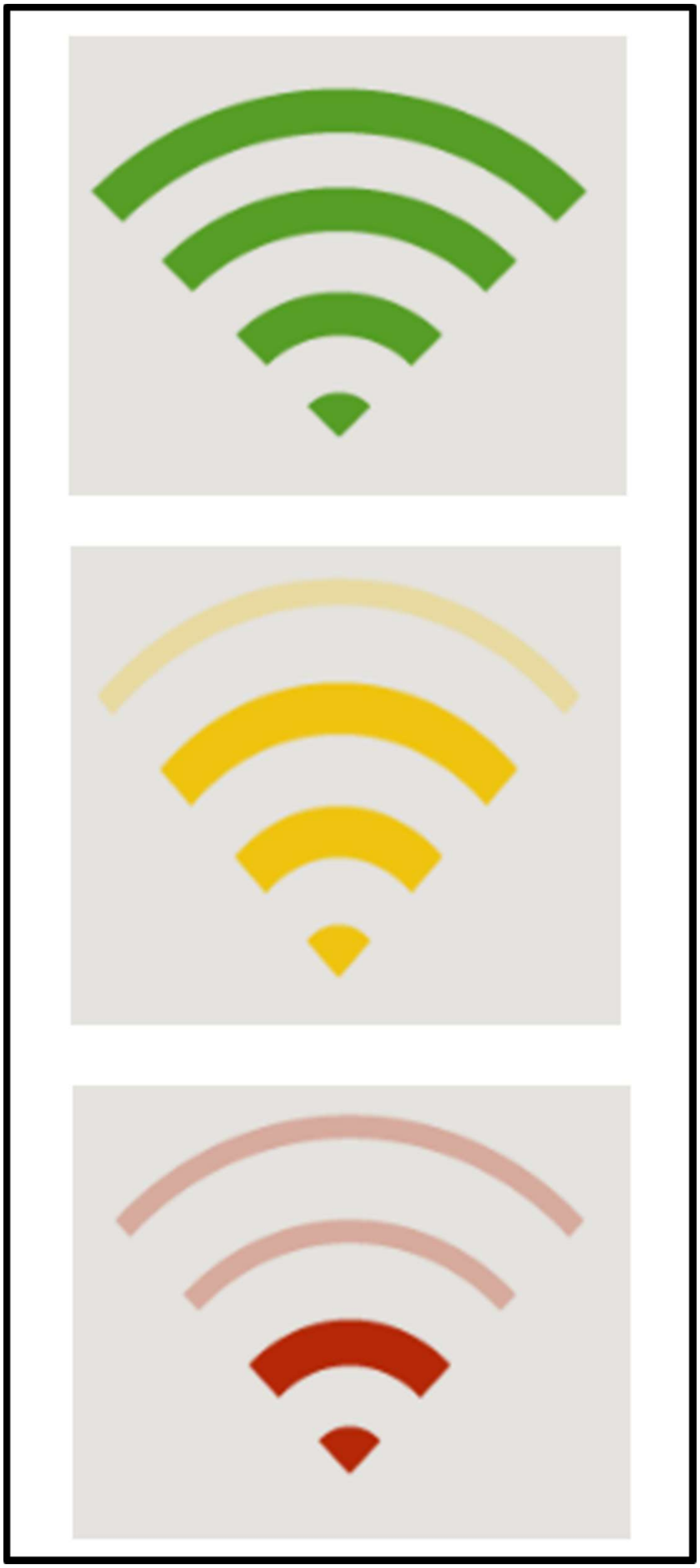

What STRESSES You?

What are your 3 BIGGEST stressors?

1. ______________________________

2. ______________________________

3. ______________________________

RELAX YOUR BODY, RELAX YOUR EMOTIONS

Relax Your Body

1. Deep Breathing: While sitting, lying down or standing, close your eyes and breathe in slowly. Let your breath out for a count of 4 seconds. Do this 10 times any time you feel tense.

2. Stretching: Practice simple stretches, such as a neck stretch. Stretch your neck by gently rolling your head in a half circle, starting at one side, then dropping you r chin to your chest, then to the other side. Be careful not to roll your head back.

3. Exercise: All kinds of physical activity – walking, running, playing sports, dancing – help to reduce stress.

4. Take a Break: Ask family members to allow you at least 30 minutes of time alone.

5. Eat Well: Reduce caffeine (in coffee, tea, soda and chocolate) and alcohol intake. Take steps to eat healthy.

Relax Your Emotions

1. Talk: Take time to talk with a friend, partner or child. Express feelings you might have been holding in.

2. Laugh: Go see a funny movie, watch a funny video, or spend time with a friend who makes you laugh.

3. Cry: Crying can be as good a release as laughing. If you haven‘t cried in a long time, try listening to sad music or watching a sad movie.

4. Read: A good book is a great escape.

5. Do Something You Love: When you enjoy yourself, whether you are dancing, going to the park, or seeing friends, you relax your emotions.

6. Write: Write things down or draw pictures about how you feel.

DECIDE Model

D = Define the problem.
You might say "I need to figure out what is bothering me."

E= Explain ways to change it.
You might say "I need to think about what I can do about my problem."

C= Choose the best option.
You might say "I need to pick the best option for me."

I = It's time to act.
You might say "I need to try out my choice."

D= Did it work?
You might say "Am I OK with the results?"

E = Explore other solutions to my problem.
You might say "If the results are not OK with me , I need to try another option."

Changing What You Do
Using the DECIDE Model

1. D = Define the problem: ____________________________

   ____

2. E= Explain ways to change it: ________________________

   ____

   ____

   ____

   ____

3. C= Choose the best option:___________________________

   ____

4. I = It's time to act: _________________________________

   ____

5. D= Did it work? ____________________________________

   ____

6. E = Explore other solutions to my problem:___________

   ____

   ____

   ____

BOOSTER SESSION #1: WEEK 26

**5-A: Welcome back & AIM Check-In**	**10 Minutes**
**5-B: PrEP use in the Future**	**5 minutes**
**5-C: Wrap-Up**	**5 minutes**
**Total**	**20 minutes**

5-A: WELCOME BACK & AIM CHECK-IN 10 MINUTES

STATE	*Welcome back! I am glad you are here today. It's been quite a while since we've met. I'd like to start by asking how your experience has been with PrEP since our last session.*
DISCUSS	Participants experience<br><br>Possible probes:<br>• Was taking PrEP harder or easier now that you are coming to clinic less often?<br>• Are you feeling more or less motivated to take PrEP than last time?<br>• Do you have any concerns about continuing PrEP for the next 3 months?
REVIEW	AIM goals with participant from last session
ASK	*Each time we've met, we discussed the AIM worksheet and your adherence goals. How are you doing with these goals?*<br><br>*Which areas of adherence are going well?*<br><br>*Which areas are not going as well?*
REVIEW	Only review areas where the participant has the most trouble.<br><br>Potential probes for Identifying Problems (I) and Making A Plan (M):<br>1. Getting to study visits<br>c. I: What causes you to miss appointments? What causes you to be late to appointments?<br>d. M: What will you do next time you miss an appointment? What will you do if you don't have transportation?<br>2. Communicating with study team<br>a. I: What causes you to not communicate with study team? (e.g., uncomfortable asking questions, visit feels rushed, etc.)<br>b. M: How might you practice communication with them? (e.g., write down questions, role play asking them)<br>3. Coping with side effects<br>a. I: Do side effects get in the way of you taking the PrEP? What have you tried to do for your side effects? Have you spoken to the study team?<br>b. M: Will you still be able to keep taking your PrEP as prescribed? What can you do to help manage side effects?<br>4. Obtaining medication and health products<br>a. I: What might cause you to run out of medicine? What might get in the way of you refilling the prescription?<br>b. M: How can you make sure you will not run out of medicine? What will help you remember?<br>5. Sticking with a daily medication schedule

	a. I: When do you seem to forget to take your PrEP? Do you take your PrEP at a time you regularly do something else? (e.g. breakfast, before bed, brushing teeth)<br>b. M: What activities can you do at the same time as you take your PrEP to help you remember?<br>6. Storing medication<br>a. I: Where do you keep your PrEP when you take it with you? Where do you keep it at home?<br>b. M: Would you be able to use a pillbox or carry case?<br>7. Reminder strategies<br>a. I: What would be helpful reminders to take your PrEP?<br>b. M: Which reminder will work best for you?<br>8. Handling slips and adherence<br>a. I: What thoughts do you think might keep you from getting back on track if you miss a dose/get off your routine? (e.g. look for all-or-nothing thinking, catastrophic thinking)<br>b. M: What could you learn from a lapse that might help you avoid another one later on? What can you do to get back on track?
REWORK	A-I-M strategies if needed to address any barriers participant may have and make new plans as needed
ASK	*Is there anything else that might get in the way of your doing any of these steps?*
ALLOW	Time for discussion

5-B: DECISIONAL BALANCE: PREP USE IN FUTURE 5 MINUTES

**STATE**	*An important component of PrEP use is continually evaluating the pros and cons of taking PrEP – and the importance of PrEP for you.*
**ASK**	*Now that you've been taking PrEP as part of this study, what do you think are the pros and cons of PrEP for people in general? Let's use a decisional balance worksheet like we did for pregnancy in a previous session.*
**HANDOUT**	Decisional Balance: Staying on PrEP
**DISCUSS**	Pros may include:<br>• HIV protection<br>• Female controlled protection<br>• Privacy of method<br>• Simple regimen<br><br>Cons may include<br>• Taking a pill daily<br>• Hassle of medical appointments<br>• Cost<br>• Stigma

**STATE**	*Thank you for working on this today. The next time we meet, we will discuss this again and make plans for your PrEP use/non-use once the study ends.*
**DISCUSS**	Any questions or concerns
**STATE**	*Thank you for being open in your discussion of PrEP today. As always, it's a pleasure continuing to work with you as part of the HERS Study.*

MY ADHERENCE GOALS

A – Adherence Goal—State It!

I – Identify problems with reaching the goal as well as what you're already doing well

M – Make a plan to overcome the problems and develop a back-up plan

(A) ______________________

(I) ______________________

(M) ______________________

(A) ______________________

(I) ______________________

(M) ______________________

(A) ______________________

(I) ______________________

(M) ______________________

(A) ______________________

(I) ______________________

(M) ______________________

DECISIONAL BALANCE: STAYING ON PREP

	PROS/BENEFITS	CONS/COSTS
TAKING PREP		
NOT TAKING PREP		

BOOSTER SESSION #2: WEEK 39

**6-A: Welcome back & AIM Check-In**	**10 Minutes**
**6-B: PrEP Plans Post-Study**	**5 minutes**
**6-C: Referrals and Wrap-Up**	**5 minutes**
**Total**	**20 minutes**

6-A: WELCOME BACK & AIM CHECK-IN 10 MINUTES

**STATE**	*Welcome back! I am glad you are here today for our last session together. It's been quite a while since we've met. I'd like to start by asking how your experience has been with PrEP since our last session.*
**DISCUSS**	Participants experience<br><br>Possible probes:<br>• Was taking PrEP harder or easier now that you are coming to clinic less often?<br>• Are you feeling more or less motivated to take PrEP than last time?<br>• Do you have any concerns about continuing PrEP for the next 3 months?
**REVIEW**	AIM goals with participant from last session
**ASK**	*Each time we've met, we discussed the AIM worksheet and your adherence goals. How are you doing with these goals?*<br><br>*Which areas of adherence are going well?*<br><br>*Which areas are not going as well?*
**REVIEW**	Only review areas where the participant has the most trouble.<br><br>Potential probes for Identifying Problems (I) and Making A Plan (M):<br>1. Getting to study visits<br>e. I: What causes you to miss appointments? What causes you to be late to appointments?<br>f. M: What will you do next time you miss an appointment? What will you do if you don't have transportation?<br>2. Communicating with study team<br>a. I: What causes you to not communicate with study team? (e.g., uncomfortable asking questions, visit feels rushed, etc.)<br>b. M: How might you practice communication with them? (e.g., write down questions, role play asking them)<br>3. Coping with side effects<br>a. I: Do side effects get in the way of you taking the PrEP? What have you tried to do for your side effects? Have you spoken to the study team?<br>b. M: Will you still be able to keep taking your PrEP as prescribed? What can you do to help manage side effects?<br>4. Obtaining medication and health products<br>a. I: What might cause you to run out of medicine? What might get in the way of you refilling the prescription?<br>b. M: How can you make sure you will not run out of medicine? What will help you remember?<br>5. Sticking with a daily medication schedule

	a. I: When do you seem to forget to take your PrEP? Do you take your PrEP at a time you regularly do something else? (e.g. breakfast, before bed, brushing teeth)<br>b. M: What activities can you do at the same time as you take your PrEP to help you remember?<br>6. Storing medication<br>a. I: Where do you keep your PrEP when you take it with you? Where do you keep it at home?<br>b. M: Would you be able to use a pillbox or carry case?<br>7. Reminder strategies<br>a. I: What would be helpful reminders to take your PrEP?<br>b. M: Which reminder will work best for you?<br>8. Handling slips and adherence<br>a. I: What thoughts do you think might keep you from getting back on track if you miss a dose/get off your routine? (e.g. look for all-or-nothing thinking, catastrophic thinking)<br>b. M: What could you learn from a lapse that might help you avoid another one later on? What can you do to get back on track?
**REWORK**	A-I-M strategies if needed to address any barriers participant may have and make new plans as needed
**ALLOW**	Time for discussion

6-B/C: PLANNING FOR PREP AFTER THE STUDY 10 MINUTES

**STATE**	*Last time that we met we talked about some general pros and cons of PrEP use, but discussed that ultimately the decision to stay on PrEP is a personal one. We used a decisional balance sheet to review the pros and cons for you. Let's look at that again*
**HANDOUT**	Decisional Balance: Staying on PrEP
**ASK**	*Are there any additional pros or cons that you would like to put into the worksheet?*<br><br>*Are there different considerations for pros/cons when you consider taking PrEP outside of the HERS study?*
**PROVIDE**	Time for participant to fill out in session
**DISCUSS**	Any questions or concerns<br><br>Probe for:<br>• PrEP access<br>• Cost coverage<br>• Clinic locations
**PROVIDE**	Referrals and information as needed for PrEP services
**STATE**	*That concludes our counseling sessions, but you will still return for one final study visit. I want to thank you for taking the time to share your experiences*

	with me. I very much appreciate the hard work and dedication that you've shown in these sessions. I wish you all the best for a healthy and happy future.

MY ADHERENCE GOALS

A – Adherence Goal—State It!

I – Identify problems with reaching the goal as well as what you're already doing well

M – Make a plan to overcome the problems and develop a back-up plan

(A)

(I)

(M)

(A)

(I)

(M)

(A)

(I)

(M)

(A)

(I)

(M)

DECISIONAL BALANCE: STAYING ON PREP

	PROS/BENEFITS	CONS/COSTS
TAKING PREP		
NOT TAKING PREP		
