# Supplementary material for: PrEP uptake, persistence, adherence, and effect of retrospective drug level feedback on PrEP adherence among young women in southern Africa: Results from HPTN 082, a randomized controlled trial
Source: PLoS Med. 2021 Jun 18;18(6):e1003670. doi: 10.1371/journal.pmed.1003670 (PMC8253429; doi:10.1371/journal.pmed.1003670)
Supplement: S4 Text — (PDF) [file pmed.1003670.s004.pdf]

Document Type: **SAP**

HPTN 082

Uptake and adherence to daily oral PrEP as a primary prevention strategy for young African women: A Vanguard Study

Statistical Analysis Plan

Statistical Center for HIV/AIDS Research and Prevention

Version 1.0, Nov 16, 2018

1. INTRODUCTION

The purpose of this SAP is to assess the acceptance rate, adherence, acceptability, and continuation of oral pre-exposure prophylaxis (PrEP) among young southern African women. It includes detailed description of Statistical Analysis related to primary and secondary endpoints in the study.

2. Study Objectives and Summary

2.1. Study description

2.1.1 Purpose: To assess the acceptance rate, adherence, acceptability, and continuation of oral pre-exposure prophylaxis (PrEP) among young southern African women.

2.1.2 Design: A Phase IV randomized multi-site prospective study to assess PrEP acceptance and adherence among HIV-uninfected young women. All women who accept open-label daily oral PrEP will be randomized 1:1 to receive enhanced adherence counselling based on feedback from observed drug levels or standard adherence support. A subset of up to ~25 women per site (maximum 75), will participate in qualitative assessments of facilitators and barriers for PrEP acceptance, adherence and continuation.

2.1.3 Study Population: HIV-uninfected women ages 16-25.

2.1.4 Study Sites: Spilhaus Clinical Research Site in Harare, Zimbabwe, the Emavundleni Research Centre in Cape Town, South Africa and Wits Reproductive Health and HIV Institute (RHI) in Johannesburg, South Africa.

2.1.5 Expected Study Size: 400 young women who accept PrEP at enrollment and up to 200 young women who decline PrEP at enrollment.

2.1.6 Study Duration: Approximately 24 months, including submissions to Institutional Review Boards (IRBs) and national drug regulatory authorities, recruitment, and 12 Months of follow-up per participant.

2.1.7 Treatment Regimen: All participants will be offered once daily oral emtricitabine 200 mg / tenofovir disoproxil fumarate 300 mg (FTC/TDF).

2.1.8 Study Visits: Screening, Enrollment, Week 4, Week 8, Week 13, Week 26, Week 39, Week52(Exit Visit).

2.1.10 Primary Objective:

- To assess the proportion and characteristics of young HIV-uninfected women who accept versus decline PrEP at enrollment.

- To assess the difference in PrEP adherence using drug levels in young women randomized to the enhanced versus standard arms.

2.1.11 Secondary Objectives:

- To assess the timing of PrEP acceptance among women who initially decline PrEP at enrollment but elect to accept PrEP during follow up.

- To assess correlates of early and delayed acceptance of PrEP, including sociodemographic factors, individual-level and partner-level characteristics, and risk practices.

- To assess correlates of PrEP adherence at Weeks 13, 26, and 52, after adjusting for study arm, such as adherence at prior study visits, sociodemographic factors, individual-level and partner-level characteristics, exposure to study-based adherence support, and risk practices.

- To assess the proportion of young women who discontinue PrEP, timing of discontinuation, and factors associated with PrEP discontinuation.

- To assess the specificity and predictive value of a PrEP readiness tool [based on the HIV Prevention Readiness Measure (HPRM) and PrEP Beliefs Measure (PBM)] to predict uptake and adherence to oral PrEP.

- To explore qualitative factors that influence women's decisions to use PrEP, to adhere to PrEP, and acceptability of PrEP in the first 3 months after PrEP acceptance.

- To compare adverse events between young women taking PrEP and young women who are not taking PrEP.

- To assess HIV incidence in those who accept PrEP compared to those who do not, and to assess the association with detectable TFV in PrEP users who acquire HIV infection during the study.

2.2. Eligibility

Young women 16-25 years old will be enrolled in this study, with a goal to enroll 125 (31%) of the 400 PrEP users in the 16-20 age range. Participants will be selected for the study according to the inclusions and exclusion criteria

Inclusion Criteria:

Young women who meet all of the following criteria are eligible for inclusion in this study:

- Female at birth
- Age 16-25 years
- Per participant report, sexually active, defined as having vaginal or anal intercourse at least once in the month prior to screening
- Literate in one or more of the study languages
- Willing and able to provide informed consent or assent (if parental consent is required per local regulations)
- If parental consent is required per local regulations, parent/legal guardian willing

and able to consent to all study procedures including HIV testing
- Able and willing to provide adequate locator information, as defined in site Standard Operating Procedures (SOPs)
- Have a score of 5 or greater on the VOICE risk score tool
- Interest in PrEP (ascertained by selected questions from the HPRM and PBM defined in the Study Specific Procedures [SSP] Manual)
- Regular access to a mobile phone with SMS capacity
- Agrees not to participate in other research studies involving drugs or medical devices for the next 12 months
- Hepatitis B virus (HBV) seronegative and accepts HBV vaccination.

Exclusion Criteria:

Young women who meet any of the following criteria will be excluded from this study:
- Planning to relocate in the next 12 months
- Has a job or other obligations that would require long absences from the area (> 4 weeks at a time) for 12 months
- Any health condition that may interfere with participation, including any debilitating or life-threatening conditions
- Currently pregnant or planning to become pregnant in the next 12 months
- Any reactive or positive HIV test at Screening or Enrollment, even if subsequent testing indicates that the person is HIV-uninfected
- Renal dysfunction (Creatinine Clearance < 60 ml/min, Schwartz Equation)
- Any reported PrEP use within the last 12 months
- Concomitant participation in a clinical trial using investigational agents, including placebo-controlled clinical trials using such agents
- Prior participation in the active arm, or current participation in any arm, of an HIV vaccine trial.
- Signs or symptoms of acute HIV infection (as described in the SSP Manual)
- Current active and serious infections which could interfere with study participation, including active tuberculosis infection, osteomyelitis, and all infections requiring parenteral antibiotic therapy (other than STIs requiring intramuscular injections of antibiotics); active clinically significant medical problems including poorly controlled cardiac disease (e.g., symptoms of ischemia, congestive heart failure), or previously diagnosed malignancy expected to require further treatment.
- Current use of ARV drugs for post-exposure prophylaxis (PEP) or completion of a PEP regimen within 4 weeks prior to Screening
- History of pathological bone fracture not related to trauma
- Known allergy/sensitivity to the study drug or its components
- Receiving ongoing therapy with any of the following: investigational ARV agents, interferon or interleukin therapy, agents with substantial nephrotoxic potential, other agents that may inhibit or compete for elimination via active renal tubular secretion (e.g., probenecid), and/or other investigational agents
- Any other condition that, based on the opinion of the site Investigator of Record (IoR) or designee, would preclude provision of informed consent, make participation in the project unsafe, complicate interpretation of outcome data, or otherwise interfere with achieving the project objectives.

3. Populations

Note: Participants who are determined during the study to have been enrolled in violation of the study protocol may be excluded from any/all analysis datasets.

- BASELINE: All women who are HIV-uninfected at enrollment will be included in this dataset. Women who are enrolled but later determined to have been HIV-infected at enrollment will be excluded from the dataset.
  This dataset will include acceptors and decliners of PrEP at baseline. Participants who have been enrolled in the study as per the inclusion criteria in section 1.1, agreed to accept PrEP and have been randomized to either standard care or Enhanced adherence counselling arm are the acceptors whereas those participants who have been enrolled as per the inclusion criteria in the study but refused to accept PrEP are the decliners.

- FOLLOW UP: Data available at all study visits will be included in this dataset. The study visits are Screening, Enrollment, Week 4, Week 8, Week 13, Week 26, Week 39, Week52(Exit Visit) (section 1.8).

- ACCEPTORS: Participants who accept PrEP and are randomized
  - Early Acceptors: Participants randomized at enrollment will be included in this dataset
  - Late Acceptors: Participants who initially declined to accept PrEP at enrollment but elect to accept PrEP during follow-up will be included in this dataset.

- PK DATA: This data will include TFV-DP collected at week 4,8,13, 26 and 52. This will be collected only among the acceptors of PrEP.

4. Statistical Data Analysis

4.1. Covariates Section

a. Sociodemographic characteristics (age, current living situation, household composition, private space for product storage):
   - Data collected: Baseline among acceptors and decliners.

   - Age: We will calculate age as the difference between date of birth and date of enrollment.
   - Race
   - Educational status
     - Currently in school
     - Ever dropped out of school
   - Marital status (Is the ppt currently married or living with her Primary partner)
   - Does her husband or primary partner provide the participant with financial and/or material support?
   - Does her husband or primary partner have any sex partners other than the participant?
   - Does the ppt have chlamydia, gonorrhea, trichomonas or syphilis?
   - Household composition, private space for product storage

- Do you have a regular place where you stay and store your things?

- Summary of Voice risk Score and its components: Each item in voice risk questionnaire is assigned points based on participants' responses. We will calculate voice risk score for each participant as a total sum of those points.

b. Partnership characteristics, primary partner's HIV status and Sexual behaviors:

-Data collected: Baseline, week 13, week 26 and end visit (week 52) among acceptors and decliners of PrEP.

- Age difference (years)
- Length of relationship
- HIV status
- Whether primary partner had sex with anyone besides her in the past 3 months
- Number of vaginal sex episodes past month
- Condom use during vaginal sex in the past month
- Anal sex episodes past month
- Condom use during anal sex in the past month

c. HIV risk perception
Data collected: Baseline, week 13, week 26 and end visit (week 52) among acceptors and decliners of PrEP.

- Chances of getting HIV in the next year
- Female friends' chances of getting HIV next year

d. HIV stigma

- Data Collected: Baseline among both acceptors and decliners of PrEP.

- Feel ashamed of using PrEP
- Feel embarrassed about using PrEP
- I am not following the 'rules' of my community if I take PrEP to prevent HIV
- People will give me a hard time if I tell them I am on PrEP
- People will judge me if I am taking PrEP
- I think I am at a greater risk for physical violence or rape if I am taking PrEP

- We will create stigma score by assigning the Likhart scale numerical values (1-5) and summing using the questions below. The score range will be 6-30. The response "prefer not to answer" will be set to missing.

e. Future orientation and aspirations; self-efficacy

-Data Collected: Baseline and week 39 among acceptors and decliners.

- I know that my life will be better in the future.
- The important people in my life tell me that I will have a successful life
- I trust that I will achieve that goal that I set for myself
- I believe that I will be successful even when there are difficulties in my life now
- I believe that the things I am doing now are preparing me for what I want in the future
- I can achieve my dreams if I focus on them

- Self efficacy score will be created by assigning the Likhart scale numerical values (1-5) based on the questions below and it ranges from 6-24.

f. Alcohol and drug use:
 - Data collected: Baseline, week 13, week 26 and end visit (week 52) among acceptors and decliners of PrEP.

  - Frequency of having a drink containing alcohol (including Zed)
  - Number of drinks on a typical day when drinking
  - Frequency of six or more drinks in one occasion
  - Ever had an alcoholic drink in the past month just before or during sex
  - Ever had drugs just before or during sex in the past month
  - Has the partner been drunk from alcohol in the past month

g. Gender-based violence (GBV) in past 12 months:

 Data collected: Baseline,week 13, week 26 and end visit (week 52) among acceptors and decliners of PrEP.

  - Partner punched slapped kicked bit you or caused you any type of physical harm
  - Partner insulted ignored or humiliated you
  - Partner forced you to have sex or touched sexually in any way that you did not want
  - Partner made you feel afraid unsafe or in danger

h. Post-traumatic stress symptoms:

 -Data collected: Baseline,week 13, week 26 and end visit (week 52) among acceptors and decliners of PrEP.

  - Have had nightmares about it or thought about it when you did not want to
  - Tried hard not to think about it or went out of your way to avoid situations that reminded you of it
  - Were constantly on guard, watchful, or easily startled
  - Felt numb or detached from others, activities, or your surroundings

i. Disclosure to peers, family members, teachers, and partner(s) about PrEP use and participation in the study :

Data collected: Baseline, week 13,week 26 and week 52 among acceptors of PrEP

- Do you plan to tell anyone about your plan to take PrEP?
- If you plan to tell anyone about your plan to take PrEP, answer “yes” or “no” for each person(s) you plan to tell in the list below.
  -Your sex partner
  -Your mother or your father
  -Your sister or your brother
  -Other family members
  -Friends
  -Neighbours
  -Nurse or doctor outside the study
  -Other persons

j. Depression score: We will calculate depression score as a sum of responses to the questions below. All these questions will be converted to the scale of 0-3 (Rarely or none of the time (less than 1 day)- All of the time (5-7 days)) and the response marked as “Prefer not to answer” will be set to missing. We will report depression sum score as a three category variable using following cutoff values by arm:
   - 0-15: None to mild depression
   - 16-26: Mild to moderate depression
   - >26: Severe Depression

   Data collected: Baseline, week 13, week 26 and end visit (week 52) among acceptors and decliners of PrEP.

   - Bothered by things that usually don’t bother me
   - Had trouble keeping my mind on what I was doing
   - Felt depressed
   - Felt that everything I did was an effort
   - Felt hopeful about the future
   - Felt fearful
   - My sleep was restless
   - I was happy
   - Felt lonely
   - Could not get going

k) Transactional sex:

Data collected: Baseline, Week 13, week 26 and end visit (week 52) among acceptors and decliners of PrEP.

It would be considered a transactional sex if a participant had sex with a man because he provided her with or she expected he would provide her with any of the following:

- Food
- clothes, shoes,accesories

- cosmetics
- cell phones
- items for children or family such as clothes,food,school fees
- transport,tickets or money for transport
- school fees or residence fees
- somewhere to stay
- cash
- other

4.2. Primary Analyses

Objective 1: To assess the proportion and characteristics of young HIV-uninfected women who accept versus decline PrEP at enrollment.

Dataset: BASELINE

Descriptive Analysis:
Proportion and characteristics of the participants will be presented by PrEP acceptance status
(Acceptors vs. Decliners). The proportion of women who accept PrEP at study enrollment, if the cap of 200 declining PrEP is not reached, will be assessed as the proportion of women who choose to accept PrEP at enrollment among the total number of women enrolled. If the limit of 200 declining PrEP is reached, we would assess the proportion who accepted PrEP at enrollment up to the time these 200 enrollments are achieved.
Following covariate characteristics (detailed above in section 4.0) assessed at baseline will be included in this analysis:

a) Sociodemographic characteristics
b) Partnership characteristics, primary partner's HIV status and Sexual behaviors
c) HIV risk perception
d) HIV stigma
e) Future orientation and aspirations; self-efficacy
f) Alcohol and drug use
g) Gender-based violence (GBV) in past 12 months
h) Post-traumatic stress symptoms
i) Disclosure to peers,family members,teachers,and partner(s) about PrEP use and participation in the study
j) Depression score

Statistical Analysis: Logistic regression will be used to assess the association of baseline characteristics of young HIV uninfected women between those who accept vs. decline PrEP at enrollment. Wald confidence limits will be computed. We will look at univariate analysis using each of the baseline characteristics described above. Multivariable model will be constructed using variables obtained after applying backward selection procedure with p-value significance criterion of 0.1.

Mathematically, we can write the model as:

$$\text{Log}\ (\text{prob}(Y_i|X_i,\ \beta_0,\ \boldsymbol{\beta})/1-\ \text{prob}(Y_i|X_i,\ \beta_0,\ \boldsymbol{\beta})) = \beta_0\ +\ \boldsymbol{\beta}\ \mathbf{X}\ +\ \dot{\varepsilon}_i$$

Y_i = 1 if the participant is an acceptor, 0 if not

β_0 = Intercept
β= Vector of Regression coefficients
X=Vector of Predictors
ε_i= Error term

Objective 2: To assess the difference in PrEP adherence using drug levels in young women randomized to the enhanced versus standard arm. We will use subset of participants who were randomized and received treatment drugs at any point in the study in our analysis.

Dataset: PK DATA

Descriptive Analysis:

We will

1) Plot the observed drug concentration by visit and arm , and overlay boxplots on the plotted points at each visit. Undetectable concentration will be displayed as LLOQ label in y-axis.
2) Plot barcharts of the proportion red, yellow and green concentrations at each of weeks 13, 26 and 52, with arm displayed side by side.

PrEP persistence is defined as time to first PrEP discontinuation, based on PrEP clinical hold, elective PrEP stop or missed visit with the assumption that no study drug remained after the date of first missed scheduled study visit.

We will create a disposition table for persistence displaying summary of following subsets of participants by arm and overall:

- Participants who have been continuously taking PrEP up to last follow up visit (week 52) in the study.
- Participants who have been discontinued from study product prior to week 4,8,13,26, before the study completion.

For each subset of participants described above in the disposition table, we will report adherence to daily PrEP:

a) Proportion of participants with TFV-DP threshold of >700 fmol/punch, 350-700fmol/punch, detectable – 350, and undetectable at Weeks 13, 26 and 52 after accepting PrEP by arm and overall.
b) Proportion of participants with detectable vs. Non-detectable DBS levels at Weeks 13, 26 and 52 among acceptors by arm and overall.

Statistical Analysis:

Analysis of adherence biomarkers:

We will analyze two binary outcomes separately with different DBS cutoffs

1) DBS concentrations of >700fmol/punch or <=700 fmol/punch
2) Detectable or Non-detectable DBS concentrations.

Two statistical approaches will be used

1) Primary

The primary assessment will compare proportion of TFV-DP levels in DBS >700 fmol/punch using logistic regression accounting for repeated measures. We will also assess detectable DBS levels using the same approach.

We will use GEE methodology with logit as the link function assuming the distribution of our outcome data is binomial. We will assume compound symmetry covariance structure in the analysis.

$$\text{Logit}(Y_{ij}) = \beta_0 + \beta_1\text{arm} + \beta_2\text{visit}$$

As a sensitivity analysis we will look at the interaction between visit and arm.

$$\text{Logit}(Y_{ij}) = \beta_0 + \beta_1\text{arm} + \beta_2\text{visit} + \beta_3\text{visit*arm} + \dot{\varepsilon}_{ij}$$

Y_{ij}= response for each subject *i*, measured at different occasions (e.g., time points), *j* = week 13, week 26 and week 52. Each response y_i for each participant is binary.
β_0 = Intercept
2) Secondary

The difference in proportion adherent (for each of the two binary outcomes) at each of weeks 13, 26 and 52 will be compared between arms using linear regression, adjusted by site.

Analysis of persistence:

We will use Kaplan Meier plots to assess time to first PrEP discontinuation for

1) ITT population
2) Per protocol : Excluding women
    a. after they discontinue PrEP due to a clinical or laboratory hold,
    b. after visits in the enhanced arm where women did not fully receive the enhanced intervention (either because DBS results were not returned before their next visit, or because drug level counselling did not correspond to the DBS drug levels from the laboratory

4.2 Secondary Analysis

Objective 3: To assess the timing of PrEP acceptance among both Early and Late Acceptors of PrEP.

Dataset: BASELINE, FOLLOW UP

Descriptive: We will create a table that shows total number of late acceptors of PrEP along with the timing of their PrEP acceptance.

Statistical Analysis:

Timing of PrEP acceptance will be assessed using the number who accept PrEP at enrollment and those who accept later during follow up. We will use Kaplan-Meier curve to assess the cumulative probability curve of time to PrEP initiation among acceptors.

Time to PrEP initiation among acceptors will be calculated as the time between their date of enrollment and the time when they consent to accept PrEP and are randomized (i.e. randomization date) to one of the arms.

Objective 4: To assess correlates of early and delayed acceptance of PrEP, including

Sociodemographic factors, individual-level and partner-level characteristics, and risk practices.

Dataset: BASELINE, FOLLOW UP

Descriptive Analysis:
Proportion and characteristics of participants will be presented by acceptance of PrEP (Early vs. late acceptors). Correlates of early and delayed acceptance of PrEP (i.e., acceptance of PrEP after enrollment) will include sociodemographic factors, individual-level and partner-level characteristics, and risk practices, as described for the primary objective, assessed at baseline and at the visit where PrEP is accepted.

Statistical Analysis: Logistic regression will be used to assess the association of baseline characteristics of young HIV uninfected women between early vs. late acceptors of PrEP. Wald confidence limits will be used.

Objective 5: To assess correlates of PrEP adherence at Weeks 13, 26, and 52, after adjusting for study arm, such as adherence at prior study visits, sociodemographic factors, individual-level and partner-level characteristics, exposure to study-based adherence support, and risk practices.

Dataset: BASELINE, FOLLOWUP, PK DATA

Descriptive Analysis: We will present PrEP adherence at Weeks 13, 26, and 52 based on TFV-DP levels by arm for each group of acceptors and overall. (Early vs. Late Acceptors)

Statistical Analysis: Correlates of PrEP adherence at Weeks 13, 26 and 52, after adjusting for arm, will be assessed using logistic regression with repeated measures among those who initiate PrEP at Enrollment and later during the follow up. Baseline and time dependent covariates will be used, and they include the following:

Outcome: PrEP Adherence is defined using TFV-DP levels in DBS (outcome)

- Primary: DBS concentrations of >700fmol/punch or <=700 fmol/punch at each visit.
- Secondary: Detectable concentrations of DBS

Following Covariates will be used (detailed in Section 4.0)

1) Partnership characteristics, primary partner's HIV status and Sexual behaviors:
2) Alcohol use and drug use
3) Number of partners
4) Age of primary partner
5) Transactional sex
6) Gender-based violence (GBV) in past 12 months:

Objective 6: To assess the proportion of young women who discontinue PrEP, timing of discontinuation and factors associated with PrEP discontinuation.

Following endpoints will be assessed:

- Date of discontinuation of PrEP during study follow-up
- Reasons for PrEP discontinuation

Dataset: BASELINE, FOLLOW UP

Descriptive Analysis: A table showing distribution of reasons for discontinuation among PrEP acceptors will be presented. We will also assess time to first discontinuation among acceptors using Kaplan Meier cumulative probability curve.

Objective 7: To assess the specificity and predictive value of a PrEP readiness tool [based on the HIV Prevention Readiness Measure (HPRM) and PrEP Beliefs Measure (PBM)] to predict uptake and adherence to oral PrEP.

Following endpoints will be assessed for the analysis of this objective:

- Acceptance of PrEP at Enrollment or during follow up
- DBS concentrations at week 13,26 and 52 among those women who accept (based on CRFs) and remain on PrEP (based on drug dispensed).
- Primary covariate of interest assessed at baseline will be PrEP readiness based on HPRM

We will use ROC methods to assess specificity and predictive value of a PrEP readiness tool to predict acceptance of PrEP among all women screened with PrEP readiness tool.

Objective 8: To explore qualitative factors that influence women's decisions to use PrEP, to adhere to PrEP, and acceptability of PrEP in the first 3 months after PrEP Acceptance. **(Not conducted at SCHARP)**

Following endpoints will be assessed for the analysis of this objective:

- Primary themes identified from serial IDIs among up to 25 PrEP users per site about reasons women accepted or declined PrEP; barriers and facilitations to PrEP pill-taking, whether being counseled about their drug levels at Weeks 4 and 8 affected their motivation and ability to subsequently adhere to PrEP (among participants randomized to receive drug
level feedback) and acceptability of PrEP.
- Barriers and facilitators to adherence as identified on counseling CRFs Qualitative factors that influence women's decisions to use, adhere to PrEP, and acceptability of PrEP for HIV prevention will be explored through analyses of themes from in-depth interviews of a subset of randomly selected participants in the first 3 months after PrEP acceptance. Within approximately 3 days of completing the interview, staff will complete a debriefing summary report to facilitate more rapid "real-time" summary and analysis of qualitative data prior to formal analysis of interview transcripts. A codebook will be created that organizes and defines codes corresponding to key themes from the interviews, and use Atlas-ti or a comparable qualitative analysis software package to code and analyze the qualitative data. A team composed of site staff and team investigators with expertise in qualitative research will code and analyze the qualitative data.

Objective 9: To compare adverse events between young women taking PrEP and young women who are not taking PrEP (Acceptors vs. Decliners).

Dataset:BASELINE, FOLLOW UP

Descriptive Analysis:
We will create a table showing the proportion of adverse events, using MeDRa coded AE data, by participants' PrEP uptake status (Acceptors vs. Decliners)

Objective 10: To assess HIV incidence in those who accept PrEP compared to those who do not, and to assess the association with detectable TFV-DP levels in PrEP users who acquire HIV infection during the study.

Dataset: BASELINE, FOLLOW UP
Descriptive Analysis:
We will create a table showing number of HIV infections, number of person years, HIV incidence and 95% CI computed using Poisson distribution by acceptors and decliners. We will also report timing of HIV infection and DBS concentration (if available) among those who become HIV infected during follow-up.
